# Dynamic L-type Ca_V_1.2 channel trafficking facilitates Ca_V_1.2 clustering and cooperative gating

**DOI:** 10.1016/j.bbamcr.2018.06.013

**Published:** 2018-06-28

**Authors:** Debapriya Ghosh, Madeline Nieves-Cintrón, Sendoa Tajada, Ingrid Brust-Mascher, Mary C. Horne, Johannes W. Hell, Rose E. Dixon, Luis F. Santana, Manuel F. Navedo

**Affiliations:** a Department of Pharmacology, School of Medicine, One Shields Avenue, University of California, Davis, CA 95616, USA; b Department of Physiology & Membrane Biology, School of Medicine, One Shields Avenue, University of California, Davis, CA 95616, USA; c Advanced Imaging Facility, School of Veterinary Medicine, One Shields Avenue, University of California, Davis, CA 95616, USA

**Keywords:** Ion channels, In vivo imaging, Vesicles, Coupled gating

## Abstract

L-type Ca_V_1.2 channels are key regulators of gene expression, cell excitability and muscle contraction. Ca_V_1.2 channels organize in clusters throughout the plasma membrane. This channel organization has been suggested to contribute to the concerted activation of adjacent Ca_V_1.2 channels (e.g. cooperative gating). Here, we tested the hypothesis that dynamic intracellular and perimembrane trafficking of Ca_V_1.2 channels is critical for formation and dissolution of functional channel clusters mediating cooperative gating. We found that Ca_V_1.2 moves in vesicular structures of circular and tubular shape with diverse intracellular and submembrane trafficking patterns. Both microtubules and actin filaments are required for dynamic movement of Ca_V_1.2 vesicles. These vesicles undergo constitutive homotypic fusion and fission events that sustain Ca_V_1.2 clustering, channel activity and cooperative gating. Our study suggests that Ca_V_1.2 clusters and activity can be modulated by diverse and unique intracellular and perimembrane vesicular dynamics to fine-tune Ca^2+^ signals.

## 1. Introduction

The L-type Ca_V_1.2 channel is expressed in many cells where it plays an indispensable role in multiple physiological processes [1]. These include regulation of gene expression, neuronal excitability, hormone secretion, cell motility and muscle contraction [2–9]. Changes in Ca_V_1.2 channel expression and function have been associated with neurodegenerative disorders, cardiac arrhythmias, hypertension and vascular complications in diabetes [9–16]. However, how a cell controls the number and spatial organization of functional membrane-associated Ca_V_1.2 channels as well as their activity remain open questions under intense investigation.

The magnitude of the Ca_V_1.2-mediated current in any given cell is the result of the channel open probability, single-channel current and the number of functional channels at the plasma membrane. The biophysical properties of Ca_V_1.2 and the mechanisms regulating channel activity have been extensively studied using electrophysiological and optical approaches [9, 17–19]. These studies have revealed that Ca_V_1.2 forms clusters of various sizes throughout the plasma membrane in different cells, including neurons, and cardiac and smooth muscle cells [15, 20–24]. Clustering of Ca_V_1.2 may contribute to regional variations in channel activity within the plasma membrane [19, 20, 22, 25, 26]. Moreover, Ca_V_1.2 clustering is required for transient functional coupling between channels that results in concerted opening and closing of adjacent Ca_V_1.2 channels (e.g. cooperative gating) [20, 21, 27–29]. This cooperative gating of Ca_V_1.2 generates zones of high Ca^2+^ influx that amplify the Ca^2+^ signal and has been suggested to play a fundamental role in cardiac EC-coupling, and persistent calcium currents in neurons [19, 21, 29, 30]. In addition to the influence of associated signaling proteins (e.g. protein kinase A, protein kinase C, calcineurin, calmodulin) [19, 28, 31], regulation of intracellular and perimembrane transport of Ca_V_1.2 along tracks of microtubules and actin filament cytoskeleton may further modulate the clustering and cooperative gating of Ca_V_1.2. Indeed, microtubules and the microtubule-associated BAR domain proteins have been implicated in directing Ca_V_1.2 to the surface membrane [32–34]. The actin crosslinking protein α-actinin was also demonstrated to stabilize and promote Ca_V_1.2 localization at the plasma membrane [35, 36]. However, the extent to which trafficking of Ca_V_1.2 via cytoskeletal networks influences channel clustering and functional behavior is unclear.

Interest in examining the dynamic movement of many ion channels has been heightened in recent years [37–44], but studies on Ca_V_1.2 are surprisingly lagging. Here, we used live-cell Total Internal Reflection Fluorescence Microscopy (TIRFM) and spinning-disk confocal imaging to directly examine the function and trafficking of Ca_V_1.2 channels expressed in tsA-201 cells, a model system often used to study Ca_V_1.2 regulation and function. We found that Ca_V_1.2 exhibits distinctive intracellular trafficking patterns in disparate vesicular structures including punctate vesicles, larger ovoid vesicles and elongated tubules. We determine that actin filaments and microtubules are both required for the dynamic trafficking of these Ca_V_1.2-containing vesicles. These Ca_V_1.2 vesicular structures exhibited either a “kiss-and-run” or “kiss-and-linger” type of interaction with the plasma membrane. Moreover, Ca_V_1.2-containing vesicles undergo constitutive homotypic fusion and fission events with each other at/near the plasma membrane and throughout the intracellular compartment requiring trafficking via both actin filaments and microtubules. This directed trafficking maintains a pool of vesicles containing channels just underneath the surface membrane that along with fusion events facilitates Ca_V_1.2 clustering, channel activity and functional cooperative gating between channels. These observations may have important implications for Ca^2+^ homeostasis in a number of physiological processes.

## 2. Materials and methods

### 2.1. Cell culture and transfection of tsA-201 cells

tsA-201 cells were obtained from Sigma-Aldrich (St. Louis, MO, USA). These SV40-transformed human embryonic kidney derived cells are a well-established cell-line model to examine the activity, trafficking and regulation of ion channels, including Ca_V_1.2, as they are easy to transfect and do not express functional endogenous Ca_V_1.2 channels as determined by optical and electrical recordings of untransfected cells [21, 27, 29, 45]. Also note that the biophysical, pharmacological, molecular and cellular properties of Ca_V_1.2 transfected in tsA-201 cells are similar to those observed for Ca_V_1.2 in native cardiac and smooth muscle [19, 22, 26, 28, 45, 46]. tsA-201 cells were cultured in Dulbecco’s Modified Eagle Medium (DMEM; Gibco-Life Technologies, Grand Island, NY) supplemented with 1× pyruvate, 1× glutamax, 8% fetal bovine serum (FBS) and 5 mM glucose (without phenol red) at 37 °C in a 5% CO_2_ incubator, and passed every 4–5 days. Cells were transfected at 60–70% confluence with 0.6 μg of DNA plasmids for the rabbit pore-forming Ca_V_1.2 subunit (α_1c_; kindly provided by Dr. William A. Catterall, University of Washington, Seattle, WA) tagged with monomeric tag-RFP (tRFP) or monomeric GFP^A206K^ in the carboxy terminus, along with 0.6 μg of the accessory subunits α_2_δ and β3 (kindly provided by Dr. Diane Lipscombe from Brown University, Providence, RI) with JetPRIME transfection reagent (Polyplus Transfection SA, NY) for approximately 6 h. In some experiments, the general endoplasmic reticulum marker Sec61β-GFP (kind gift from Dr. Eammon J. Dickson, University of California Davis) or the Golgi marker glycosyltransferases-GFP (Golgi-GFP BacMam 2.0, Thermo Fisher) was co-transfected with the channel in tsA-201 cells. For Ca_V_1.2 sparklet experiments, cells were also co-transfected with the protein kinase C α (PKCα) isoforms as previously described [26]. Cells were seeded on 25 mm no. 1 coverslips (Thermo Fisher Scientific, Waltham, MA) for experiments in either a Warner Instruments QR Imaging Chamber or custom-made chambers with laminar flow. For some experiments, transfected cells were pre-treated with nocodozole (10 μM), cytochalasin D (cyt-D; 10 μM) or both compounds simultaneously for 6 h before experiments. All experiments were performed at room temperature.

### 2.2. Spinning-disk confocal and TIRF live-cell imaging

High resolution 4D recordings of tsA-201 cells expressing fluorescently tagged Ca_V_1.2 and accessory subunits were performed using an Andor Revolution spinning-disk confocal system coupled to an Olympus iX-81 inverted microscope equipped with an Olympus UApo N 100× oil immersion TIRF lens (NA, 1.49), an Andor iXon EMCCD Ultra camera, and 488 nm and 561 nm lasers with appropriate excitation and emission filters. The system was controlled with the Andor IQ software. Confocal z steps (0.5 μm) encompassing the volume of the cell over time were acquired for 3D reconstruction. The acquisition rate, exposure time, number of z steps, and laser power were kept constant for all images recorded. For imaging of two fluorophores in the same sample, cells were imaged sequentially using an appropriate dual bandpass filter to eliminate any overlapping emission. Transfected cells were imaged using a physiological saline solution (PSS) containing (in mM): 134 NaCl, 6 KCl, 1 MgCl_2_, 2 CaCl_2_, 7 _D_-glucose, 10 HEPES pH to 7.4 with NaOH.

TIRF imaging was performed using several TIRF microscope systems depending on the experiment. To image dynamic movement of tRFP labeled Ca_V_1.2 at or near the membrane of tsA-201 cells and for Total Internal Reflection – Fluorescence Recovery after Photobleaching (TIR-FRAP) experiments, we used a Leica DMI6000 B TIRF microscope equipped with a Leica oil-immersion HC PL Apo 160× (NA, 1.43) TIRF objective, an Andor iXon3 EMCCD camera and a 532 nm laser and appropriate excitation and emission filters. Images were collected using the same penetration depth and laser power at a frame rate of 5 Hz for 1000 frames using the Leica Application Suite software. The incident angle used for these experiments afforded an evanescent wave with an expected decay length constant of ~100 nm. For these experiments, coverslips were mounted on glass depression slides (neoLab, Heidelberg, Germany) with PSS buffer and sealed with Twinsil (Picodent, Wipperfürth, Germany). The bleaching protocol was as previously described [39], and consisted of 50 exposure sequences with the 532 nm laser set at 4% of its maximal power, followed by 50 exposure sequences with laser power set at 100%, and culminating with a post-bleaching recording with laser power back at 4% power. Analysis of concentric bands was performed using a custom ImageJ plugin as previously described [38, 39]. The plugin generates 10 concentric bands equally spaced based on the freeform ROI marking the boundary of the cell.

Ca^2+^ sparklet experiments (see below) were performed using a through-the-lens TILL Photonics TIRF system built around an Olympus iX-70 inverted microscope equipped with an ApoN 60× oil immersion (NA, 1.49) TIRF objective, 2 laser lines (491 nm and 563 nm) with corresponding excitation and emission filters, and an Andor iXon EMCCD camera. The system was controlled with TiLLvisION software. For stepwise photobleaching experiments and to visualize the dynamic movement of Ca_V_1.2-GFP, we used an Olympus cellTIRF system coupled to an Olympus iX-81 inverted microscope equipped with an Olympus UApo N 100× oil immersion TIRF lens (NA, 1.49), 2 laser lines (488 nm and 561 nm) with corresponding excitation and emission filters, and a Photometrics Prime 95B sCMOS camera.

### 2.3. Quantification of compartment size

Analysis was performed using ImageJ (NIH). The size of Ca_V_1.2-containing structures was determined by fitting the fluorescence intensity profile of the crosssection of Ca_V_1.2 compartments with a Gaussian function. The full-width at half maximum (FWHM) for each curve represents the diameter of the vesicle. The radius (R) of the vesicle was thus given by FWHM/2. These data were confirmed using fluorescence beads of known diameter (100 nm). The width of the tubular structures was measured in similar fashion [47]. The length of the tubules was determined by measuring their total visible length.

### 2.4. Movement tracking with Imaris

Imaris software (Bitplane, Switzerland) was used to track the movement of vesicles and for construction of trajectory map [48]. Ca_V_1.2-containing structures were detected in 4D images after thresholding. The “Spots Module” of Imaris was used to automatically detect Ca_V_1.2-containing structures with a maximum spot diameter of 1.4 μm. Detected spots were then filtered based on “Quality” with “Background Subtraction” parameters (described as the intensity at the center of the spot in the Gaussian-filtered channel at which the spot was detected, minus the intensity of the original channel that has been Gaussian-filtered by 8/9 of the spot radius). Visual inspection was performed to confirm correct detection of structures. The maximum permissible gap length for re-detection of the same structure temporarily lost was set to 3 frames. The selected structures were then tracked automatically over time based on a built-in algorithm of autoregressive motion in Imaris, which generated trajectories for Ca_V_1.2-containing structures, along with several additional parameters including length of the trajectory, displacement, speed and duration of the event being tracked. Trajectory outputs were then visually inspected and edited as necessary to correct for tracking errors.

### 2.5. Mean squared displacement analysis

The nature of the motility of Ca_V_1.2-containing structures was assessed by calculating mean square displacement (MSD) curves plotted over time. MSD for each trajectory was calculated using the MSD analyzer from Imaris Open [49]. Trajectories with at least 8 time points, each of which was an average of at least 5 intervals, were arranged into three different groups using a custom sorting algorithm written in Matlab (Mathworks, Natick, MA) with the following three equations:

$${\text{Directed motion + Diffusion: < r}}^{2}\geq {\text{ v}}^{\text{2}}{\text{t}}^{2}+6\text{Dt}$$

$${\text{Anomalous diffusion: < r}}^{2}\geq 6{\text{Tt}}^{\text{α}};\text{ α}<1$$

$${\text{confined diffusion: < r}}^{2}\geq {\text{r}}_{\max}{}^{2}\left(1-\exp\left(-{\text{T}}_{\text{c}}\text{t}/{\text{r}}_{\max}{}^{2}\right)\right)$$

where v is the velocity of active motion, D is the diffusion coefficient, Τ is the transport coefficient, α is the anomalous diffusion exponent, r_max_ is the radius to which diffusion is constrained, and Τ_c_ is the transport coefficient for confined diffusion. All values were constrained between 0 and 1. Trajectories were further sorted according to the following rules: If the best fit to a given trajectory was diffusion plus flow, but the velocity of that trajectory was slower than 0.01 μm/s, it was considered pure diffusion, unless the diffusion coefficient was slower than 0.001 μm/s in which case it was counted as a stationary particle. If the best fit was sorted as anomalous diffusion, but the diffusion coefficient was larger than 0.9, it was considered diffusion, and if the transport coefficient was < 0.001 μm/s, it was counted as a stationary particle. Finally, in the case of confined diffusion, if the maximum radius of the compartment was smaller than 0.5 or the transport coefficient was smaller than 0.001 μm/s, it was considered stationary. It is important to note that in unique instances where tracks with pure diffusion were interrupted by a period of directed motion, our sorting algorithm labeled these tracks as anomalous diffusion if the time of the MSD was larger than a quarter of the track time. Under treatment conditions, we couldn’t always restrict the MSD to less than a quarter of the track time. Therefore, some of the particles labeled as exhibiting anomalous diffusion may have small intermittent periods of directed motion, which we confirmed by looking individually at some of these tracks.

### 2.6. Stepwise photobleaching

tsA-201 cells transfected with Ca_V_1.2-RFP or Ca_V_1.2-GFP were fixed in 4% paraformaldehyde (10 min) and imaged using the Olympus cellTIRF system described above using a 100×/1.49 NA objective. Cells were illuminated with 561 or 488 nm laser light at 5% power and 200 ms exposure and image stacks of > 2000 images were acquired. The ImageJ plugin Time Analyzer V3 was used to select regions of interest (ROI) with 20-pixel diameter. The intensity profile within each ROI was plotted over the entire image stack, after which bleaching steps were manually counted. In some experiments (Fig. 7), TIR-FRAP [38, 39, 50] was performed before cells were fixed. Stepwise photobleaching experiments were then performed as described above.

### 2.7. Electrophysiology

We used the conventional whole-cell patch-clamp technique to control membrane voltage and record macroscopic currents with Ba^2+^ or Ca^2+^ as the charge carrier using an Axopatch 200B amplifier and Digidata 1440 digitizer (Molecular Devices) in tsA-201 cells transfected with Ca_V_1.2, β_3_, α_2_δ and PKCα. Data were sampled at 20 kHz and digitally filtered at 2 kHz. The pipette solution contained (in mM) 87 Cs-aspartate, 20 CsCl, 1 MgCl_2_, 5 MgATP, 10 EGTA, 10 HEPES adjusted to pH 7.2 with CsOH. The extracellular solution contained (in mM) 115 NaCl, 5 CsCl_2_, 20 BaCl_2_ or 20 CaCl_2_, 1 MgCl_2_, 10 _D_-glucose, 10 HEPES, adjusted to pH 7.4. Pipettes were pulled from borosilicate capillary glass using a micropipette puller (mode P-97, Sutter Instruments), and polished to achieve a resistance ranging from ~3–5 MΩ. Once a GΩ seal was made and successful conversion to the whole-cell configuration was achieved, cells were depolarized for 500 ms from the holding potential of −70 mV to 0 mV or for 300 ms from the holding potential of −70 mV to voltages ranging from −80 to +70 mV to record the Ba^2+^ current or Ca^2+^ current, respectively, associated with Ca_V_1.2 activity. Data were analyzed offline using pCLAMP 10 software. For Ca_V_1.2 sparklet experiments, the extracellular solution was replaced with one containing (in mM) 120 NMDG, 5 CsCl, 20 CaCl_2_, 1 MgCl_2_, 10 _D_-glucose, 10 HEPES adjusted to pH 7.4 with HCl after establishing of the whole-cell configuration.

### 2.8. Ca_V_1.2 sparklet recordings

Ca_V_1.2 sparklet images were recorded using the TILL Photonics TIRF system described above. Images were acquired at 100 Hz. For these experiments, tsA-201 cells were transfected with Ca_V_1.2, β_3_ and α_2_δ auxiliary subunits plus PKCα as cooperative gating of Ca_V_1.2 is highly dependent on this kinase [26]. To increase the driving force for Ca^2+^ entry necessary to record quantal Ca^2+^ sparklet events, cells were patch clamped in the whole-cell configuration at −70 mV while being perfused with 20 mM external Ca^2+^ as previously described [22, 25, 26, 29, 51]. Ca_V_1.2 sparklets were recorded in cells treated with 1 μM thapsigargin to eliminate any Ca^2+^ release event from intracellular stores. Submembrane Ca^2+^ events (e.g. Ca_V_1.2 sparklets) were monitored in cells dialyzed through the patch pipette with the relatively fast Ca^2+^ indicator Fluo-5F (200 μM) and an excess of the slow, but high affinity non-fluorescent Ca^2+^ buffer EGTA (10 mM). The objective of this combination of indicator and EGTA is to facilitate that the faster Ca^2+^ indicator binds to Ca^2+^ first, thus producing a fluorescent signal. The duration of this signal will be limited as the slower but high affinity Ca^2+^ chelator EGTA buffers Ca^2+^ away. This maneuver will restrict the fluorescent signal to the Ca^2+^ entry site [52]. Ca_V_1.2 sparklets were identified and analyzed using custom software written in MATLAB as previously described [22, 25, 26, 29, 51].

### 2.9. Coupled Markov chain model

The strengths of cooperative gating between single Ca_V_1.2 channels was determined from individual Ca_V_1.2 sparklet sites using a binary coupled Markov chain model previously described [53] and subsequently implemented by our group using MATLAB [19–21, 27–29]. The activity of Ca_V_1.2 sparklets was modeled as a first order, discrete Markov chain, and the Markovian transition matrix was estimated from sparklets records and their corresponding channel opening time courses using the built-in Hidden Markov parameter estimation function in MATLAB. The program assigns a dimensionless coupling coefficient (κ) that ranges between 0 for fully uncoupled channels to 1 for fully coupled channels.

### 2.10. FM 1–43 experiments

tsA-201 cells were transfected with Ca_V_1.2-RFP, α_2_δ and β_3_ subunits. Dual channel 4D recording using spinning-disk confocal microscopy was performed using 488 and 561 laser lines. Cells were continuously perfused with buffered saline containing (in mM) 134 NaCl, 6 KCl, 1 MgCl_2_, 2 CaCl_2_, 7 Glucose monohydrate, 10 HEPES; pH of 7.4 (with NaOH). FM 1–43 (1 μM) dye was perfused after approximately 10 time points of the 4D volume recording, which continued over several minutes in the presence of FM 1–43 dye. The recorded images were median filtered and displayed as maximum intensity Z stack projections over time.

The fluorescence intensity quantification displayed in Fig. 2B was performed using Fiji open source software. Time-series images were split into respective 561 and 488 channels. We tracked vesicle movement and quantified their fluorescence intensity in the 561 channel. Next, the same ROI was analyzed in the corresponding 488 channel. Finally, we normalized the measured fluorescence intensity values by making the highest value 100 and the lowest value zero. To make the quantification displayed in Fig. 6, 2-channel time series images were split to yield individual red and green channel images. An ROI was then drawn encompassing the plasma membrane and perimembrane space on a frame at a very early stage in FM-dye application where only the plasma membrane was clearly marked by the dye. The second ROI was positioned in the intracellular space of the cell. The same ROIs were imposed on all other frames in the time series. The fluorescence intensities in the ROIs was measured and normalized as above.

### 2.11. Quantification of fusion and fission events

Quantification of homotypic fusion and fission events between Ca_V_1.2-containing structures was done with the TrackMate plugin in ImageJ. Briefly, stacks of tiff images were opened in ImageJ, and the TrackMate plugin was launched. The ‘Laplacian of Gaussian detector’ was used for tracking events of interest. The detector spots were filtered accordingly to ‘quality’ and ‘signal to noise ratio’. The detection of the structures was visually confirmed, and the thresholds and filters were adjusted accordingly. A ‘Linear Assignment Problem’ tracker, which allows detection of merging (e.g. fusion) and splitting (e.g. fission) events, was then used to track vesicle movement. The number of tracks undergoing fusion and fission events was then quantified for each control and treated cell recording.

### 2.12. Chemicals and statistics

All chemical reagents were from Sigma-Aldrich (St. Louis, MO) unless otherwise stated. Data were analyzed using GraphPad Prism software and expressed as mean ± SEM. Data were assessed for potential outliers using the GraphPad Prism Outlier Test and for normality of distribution using the Shapiro-Wilk or KS normality tests. Statistical significance was then determined using appropriate paired or unpaired Student’s *t*-test, nonparametric tests or One-way analysis of variance (ANOVA) for multiple comparisons with appropriate post hoc test. *P* < 0.05 was considered statistically significant (denoted by * in figures).

## 3. Results

### 3.1. Ca_V_1.2 undergoes dynamic perimembrane and intracellular transport in vesicular structures

**Fig. 1.**
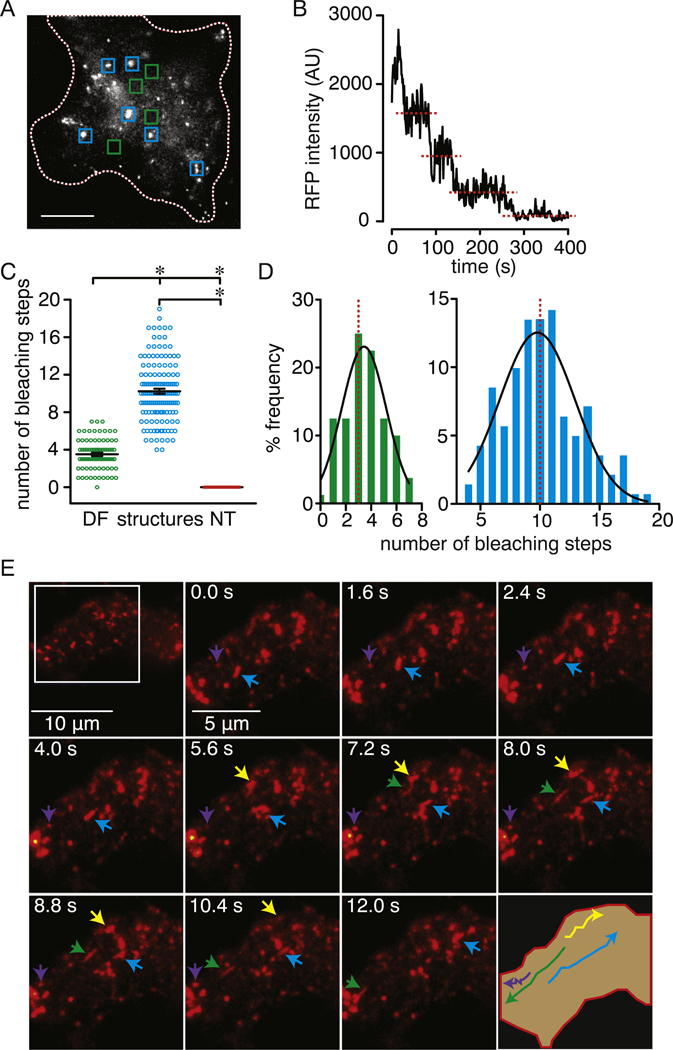
Heterogeneous distribution and dynamic perimembrane movement of Ca_V_1.2. A) Representative TIRFM image of a fixed tsA-201 cell expressing Ca_V_1.2-RFP. The blue and green squares highlight areas with clearly identifiable Ca_V_1.2 structures and area of the cell footprint with diffuse fluorescence (DF), respectively. These areas were used for analysis. The white dotted line outlines the boundary of the cell footprint. Scale bar = 10 μm. B) Representative time course of bleaching steps for Ca_V_1.2-RFP associated fluorescence. C) Scatter plot of the number of bleaching steps obtained from plasma membrane regions with diffuse fluorescence (DF; n = 80 regions from 6 cells), Ca_V_1.2 structures (structures; n = 141 regions from 6 cells) and from untransfected cell (NT; n = 100 regions from 6 cells). Data are shown as mean ± SEM. *P < 0.05. One-way ANOVA with Tukey’s multiple comparison tests. Significance was compared between data as specified. D) Frequency distribution of bleaching steps between PM (green histogram) and structure (blue histogram) regions. Histograms were fit using a single Gaussian curve (black lines). The dotted red lines denote the median of the distribution (3 for PM and 10 for structures). E) TIRFM images at consecutive intervals depicting the perimembrane movement of Ca_V_1.2 structures in vehicle-treated tsA-201 cells transfected with Ca_V_1.2-RFP. Arrowheads point to track of different Ca_V_1.2 structures. The *lower right corner* image illustrates the movement tracks of the respective structures highlighted by the arrowheads.

Experiments were performed using tsA-201 cells transfected with Ca_V_1.2 fused to monomeric tRFP (Ca_V_1.2-RFP) or monomeric GFP^A206K^ (Ca_V_1.2-GFP) along with β_3_ and α_2_δ auxiliary subunits. Nifedipine-sensitive Ba^2+^ currents (*I*_Ba_) were readily recorded from these cells (Fig. S1A). The distribution of Ca_V_1.2 proximal to the surface membrane of fixed tsA-201 cells was examined using TIRFM [18, 54]. Whereas untransfected cells did not show any fluorescence (Fig. S1B), Ca_V_1.2-RFP/ GFP-associated fluorescence in transfected cells was diffused throughout the surface membrane with distinct areas of high fluorescence intensity in discrete structures (Fig. 1A and Fig. S1C).

Stepwise photobleaching analysis was used to determine the number of Ca_V_1.2-RFP channels in the perimembrane region [55]. This analysis revealed distinct populations with stepwise decreases in fluorescence in those areas containing discrete Ca_V_1.2 structures (blue squares; structures) compared to the areas of the plasma membrane with a more diffuse fluorescence pattern (green squares; DF) (Fig. 1A–D). No stepwise decrease in fluorescence was observed in untransfected tsA-201 cells (NT; Fig. 1C). The mean number of bleaching steps was 3.5 ± 0.2 in regions with diffused fluorescence versus 10.2 ± 0.3 in regions with discrete Ca_V_1.2 structures (Fig. 1C and D). Comparable results were observed in cells expressing Ca_V_1.2-GFP (Fig. S1C and S1D), thus corroborating that the Ca_V_1.2 distribution pattern is similar irrespective of the fluorescence reporter. These results are consistent with previous studies [20–22, 26, 29], and suggest a heterogeneous distribution of Ca_V_1.2 with channel clustering tending to occur at discrete sites at/near the surface membrane.

To examine whether the discrete Ca_V_1.2-RFP- (and Ca_V_1.2-GFP-) associated structures observed at/near the surface membrane of tsA-201 cells are mobile, we performed live-cell TIRFM imaging. Video 1 demonstrates that the discrete Ca_V_1.2-RFP structures move dynamically throughout the footprint of this vehicle-treated cells (Fig. 1E). Similar results were found in cells transfected with Ca_V_1.2-GFP (Fig. S1E) or with both Ca_V_1.2-RFP and Ca_V_1.2-GFP (Fig. S1F). The discrete Ca_V_1.2 structures are mostly circular in shape with a smaller percentage displaying a tubular contour (Fig. S2A–B). The radius of the circular Ca_V_1.2 structures ranged from 137 nm to 1362 nm with an average of 341 ± 14 nm and a median value of 254 nm (Fig. S2C–E). A frequency distribution histogram of the circular structures revealed the presence of two distinct populations with the bulk of those observed being small punctate structures with a radius ranging from 140 nm to 300 nm, whereas larger structures (referred to as ovoids) had radi of > 350 nm (Fig. S2E). The transverse and longitudinal mean radii of the tubular compartments were 231 ± 19 nm and 2500 ± 190 nm, respectively (Fig. S2F).

**Fig. 2.**
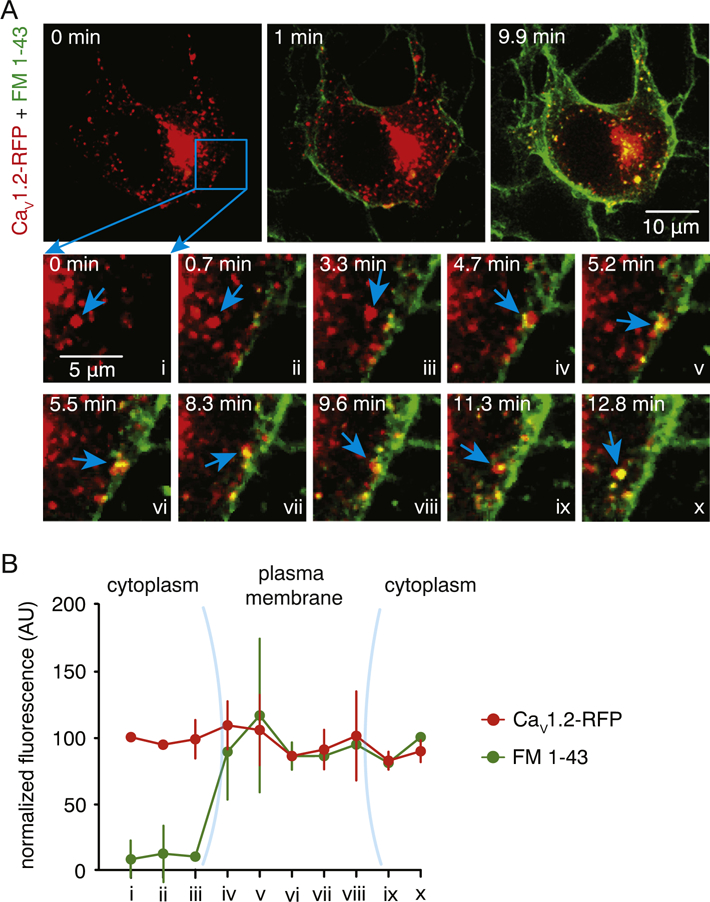
Interaction of Ca_V_1.2 structures with plasma membrane. A) Representative dual-color spinning-disk 3D images of a vehicle-treated tsA-201 cell expressing Ca_V_1.2-RFP before (0 min) and after (1 and 9.9 min) application of fluorescent plasma membrane dye FM 1–43 dye. Lower panels depict enlarged time lapse illustrations of the area highlighted by the blue square. The blue arrows point to a Ca_V_1.2 structure that approaches the plasma membrane, then uptakes the FM 1–43 dye and moves to the interior of the cell. B) Normalized FM 1–43 (green) and Ca_V_1.2 (red) fluorescence intensity (AU) of Ca_V_1.2 structure highlighted by the blue arrowhead in panel A over time. Data are shown as mean ± SEM (n = 6 cells).

We next examined whether the discrete Ca_V_1.2 structures were of vesicular origin and present not only throughout the intracellular space but also would physically interacting with the plasma membrane. We performed sequential 4D recordings of Ca_V_1.2-RFP in vehicle-treated cells following perfusion of the fluorescent membrane probe FM 1–43 excited with 488 nm light [56, 57]. Note that robust staining of the plasma membrane was observed shortly after dye perfusion (Video 2 and Fig. 2A). The representative movies in Videos 2 and 3 show the presence of highly mobile Ca_V_1.2 structures throughout the intracellular space of vehicle-treated cells (see also Fig. 2A). This was never observed in untransfected cells (Video 4, arrowheads). Movement of these structures was distinctly independent of the ER (Video 5 and Fig. S3A–B). Although Ca_V_1.2 structures originate from the Golgi suggesting a classic Golgi secretory route, the lack of Golgi marker fluorescence in the structures at the periphery of the cell indicate that they are independent of the Golgi body (Video 6 and Fig. S3C–D).

Analysis of simultaneous 4D recordings revealed that Ca_V_1.2 structures (red) can approach the plasma membrane (Fig. 2Ai–Aiii and B) where they can linger while readily taking up the FM 1–43 dye (green; Fig. 2Aiv–Avi and B; Video 7). This results in the formation of yellow structures (highlighted by the blue arrow in Fig. 2A). These yellow structures formed by the exchange of the FM 1–43 dye from the surface membrane to the Ca_V_1.2 structure subsequently moved to the interior of the cell, suggesting endocytosis of Ca_V_1.2 structures (Fig. 2Avi–Ax and B). These results support the view that these Ca_V_1.2 structures are vesicular in nature and can physically interact with the plasma membrane. Taken together, our results suggest that Ca_V_1.2 channels contained within vesicular structures undergo dynamic perimembrane and intracellular transport.

### 3.2. Cytoskeleton-mediated diverse and dynamic intracellular movement of Ca_V_1.2

**Fig. 3.**
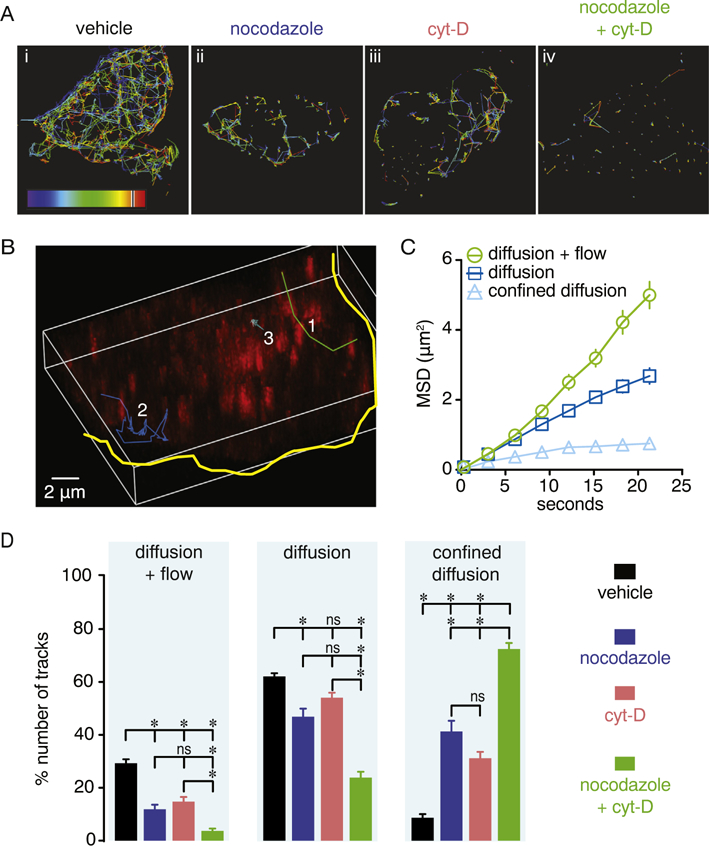
Dynamic intracellular movement of Ca_V_1.2 vesicles requires actin filaments and microtubules. A) Representative trajectories of the intracellular movement of Ca_V_1.2 vesicles reconstructed from 4D recordings of vehicle-treated, nocodazole (10 μM), cytochalasin-D (10 μM) or nocodazole + cytochalasin-D (10 μM each) -treated cells. B) Representative 3D image of reconstructed trajectories from three distinct intracellular movement tracks (1–3) exhibited by Ca_V_1.2 vesicles. The yellow solid line outlines the cell boundary. Trajectory 1 (green line) exemplifies diffusion + flow movement, trajectory 2 (blue line) represents diffusion, and trajectory 3 (light blue) illustrates confined diffusion. C) Graph illustrating average MSD over time of Ca_V_1.2 vesicles in vehicle-treated cells for the three movement patterns identified (n = 6 cells, > 80 tracks per movement pattern). Data shown as mean ± SEM. D) Bar plots depict percentage of the total number of trajectories exhibiting a specific movement pattern in vehicle-treated (n = 15 cells), nocodazole (n = 16 cells), cytochalasin-D (n = 14 cells) or nocodazole + cytochalasin-D (n = 7 cells) -treated cells. Data are shown as mean ± SEM. *P < 0.05. Kruskal-Wallis test. Significance was compared between data as specified.

Consistent with the results above, single particle tracking within our 4D recordings revealed dynamically diverse mobility patterns of intracellular Ca_V_1.2 vesicles in vehicle-treated cells (Fig. 3Ai and B and Fig. S4A). Mean square displacement (MSD) analysis obtained from trajectories of these vesicles in vehicle-treated cells (Fig. 3C) revealed that ~29% of the observed structures exhibit constitutive directional movement (diffusion + flow), ~62% show diffusional movement and ~8% were largely stationary and/or displayed confined diffusion movement (Fig. 3D).

We next examined whether the movement of Ca_V_1.2 vesicles required intact microtubules and/or actin filaments. For this, tsA-201 cells transfected with Ca_V_1.2-RFP were treated with either 10 μM nocodazole or 10 μM cytochalasin-D (cyt-D) for 6 h to depolymerize microtubules or actin, respectively [58, 59]. We avoided prolonged treatment of > 6 h with any of these drugs as it may result in unwanted changes in cell function, apoptosis or cells of poor quality for experimental analysis, although more harsh conditions have been employed previously [32]. Four dimensional recordings and single particle tracking analysis showed that the individual nocodazole (Video 8) and cyt-D (Video 9) treatment significantly reduced the intracellular mobility pattern of Ca_V_1.2-RFP vesicles (Fig. 3Aii–Aiii). Exposure to either drug decreased all the measured movement parameters (Fig. 3Aiv and Fig. S4B–F). Interestingly, displacement, maximum speed and mean speed were more affected by the cyt-D compared to the nocodazole treatment. Yet, the combinatorial treatment with both drugs appeared to have a synergistic effect on Ca_V_1.2 vesicular movement (Fig. 3A and Fig. S4C, D–E; Video 10).

MSD analysis showed that compared to vehicle-treated cells, depolymerization of either microtubules or actin filaments significantly decreased directional (e.g. diffusion + flow) and diffusional movement while increasing the number of Ca_V_1.2-RFP vesicles undergoing stationary or confined diffusion motion (Fig. 3D). Simultaneous treatment of cells with nocodazole and cyt-D (10 μM each drug) exacerbated the reduction in vesicle movement with the majority of the Ca_V_1.2 positive structures being either stationary or exhibiting confined diffusion (Fig. 3D). This spatially restricted motion aligns with the significantly prolonged tracking duration of Ca_V_1.2-RFP vesicles observed in Fig. S4G. These results suggest that the intracellular mobility of Ca_V_1.2 in tsA-201 cells is diverse, dynamic and highly dependent on both microtubules and actin filaments.

### 3.3. Constitutive perimembrane turnover of Ca_V_1.2 channels

**Fig. 4.**
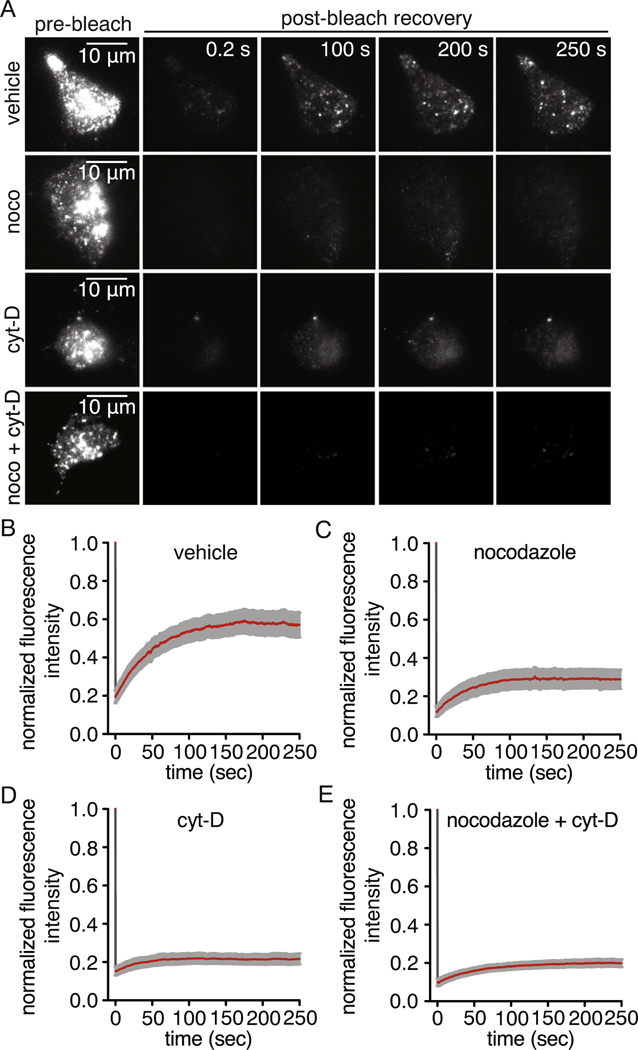
Cytoskeleton-dependent constitutive transport of Ca_V_1.2 vesicles to the perimembrane region. A) Representative TIRFM images of pre-bleach and post-bleach of Ca_V_1.2-RFP fluorescence in vehicle-treated (n = 17 cells), nocodazole (10 μM; n = 13 cells), cytochalasin-D (10 μM; n = 15 cells) or nocodazole + cytochalasin-D (n = 8 cells) -treated cells. Time course of fluorescence recovery of Ca_V_1.2-RFP following photobleaching normalized to the pre-bleach value in (B) vehicle-treated, (C) nocodazole, (D) cytocholasin-D or (E) nocodazole + cytochalasin-D -treated cells. Data are shown as mean ± SEM.

To further determine whether Ca_V_1.2 channels are present in dynamic vesicular structures that constitutively approach the plasma membrane, Total Internal Reflection – Fluorescence Recovery after Photobleaching (TIR-FRAP) [38, 39, 50] was performed on Ca_V_1.2-RFP expressing tsA-201 cells. This TIRFM configuration selectively bleaches Ca_V_1.2-associated fluorescence in the perimembrane region to facilitate visualization of Ca_V_1.2 movement to this area. Dynamic movement of Ca_V_1.2 vesicular structures was observed during pre-bleaching recordings, as before (Video 11). Following the bleaching step, Ca_V_1.2 vesicular structures constitutively repopulated the perimembrane region in vehicle-treated cells (Video 11). Analysis of fluorescence intensity when recovery was stable at 250-second post-bleach yielded a maximal recovery of 57 ± 5% of the pre-bleach fluorescence level (Fig. 4A–B). The observed level of Ca_V_1.2 recovery is comparable to that previously observed for several TRP channels [38, 39] and neuronal ATP-gated P2X3 receptors within the same time period [60]. To examine whether microtubules and actin are necessary for trafficking of Ca_V_1.2, TIR-FRAP was performed in cells treated with nocodazole, cyt-D or both compounds. Depolymerization of microtubules, actin filaments or both cytoskeletal elements simultaneously limited the perimembrane recovery of Ca_V_1.2 to 29 ± 4%, 21 ± 2% and 19 ± 2% of the prebleach value, respectively (Fig. 4A–E). These results suggest that both microtubules and actin filaments are important determinants of Ca_V_1.2 mobility to the perimembrane region.

To assess the modes of Ca_V_1.2 channel recovery, exponential time constants of the post-bleach fluorescence recovery were computed for 10 concentric and equally spaced bands encompassing the cell footprint from the periphery (band 1) to the center (band 10) (Fig. S5A) [38, 39]. In this analysis, homogenous fluorescence recovery over the total footprint of the cell would indicate that both vesicular transports to the plasma membrane and lateral diffusion of channels from non-bleached areas of the plasma membrane contributes to the final post-bleach recovery. In contrast, fluorescence recovery that occurs at a faster rate from the sides followed by movement towards the center of the footprint of the cell would suggest an increased contribution of lateral diffusion and impaired Ca_V_1.2 vesicular trafficking. In this latter case, Ca_V_1.2-associated fluorescence will recover faster in the peripheral zone of the footprint compared to the central zone.

In vehicle-treated cells, all the bands had variable but comparable time constants of fluorescence recovery with neither center nor periphery lagging behind the other in post-bleach fluorescence recovery (Fig. S5B). The subtle, shallow negative gradient of the best-fit line suggests that vesicular movement of Ca_V_1.2-RFP channels from inside the cells towards the plasma membrane dominates the recovery of Ca_V_1.2 fluorescence in vehicle-treated cells. In cells treated with either nocodazole or cyt-D (Fig. S5C–D), a shallow yet, positive gradient of the best-fit line of the time constants of fluorescence recovery was observed, thus suggesting an increased contribution of lateral diffusion of channels in the plasma membrane from outside the bleached area to the fluorescence recovery profile. However, cells treated simultaneously with both drugs showed a steeply positive best-fit line of the time constants of fluorescence recovery (Fig. S5E). This result suggests that fluorescence recovery after disruption of both microtubules and actin filaments is driven by lateral diffusion of channels. Altogether, and in agreement with our 4D data, these results indicate that both microtubules and actin filaments contribute to constitutive trafficking of Ca_V_1.2 to and from the plasma membrane.

### 3.4. Cytoskeleton-dependent distinct perimembrane behavior of Ca_V_1.2 vesicles

**Fig. 5.**
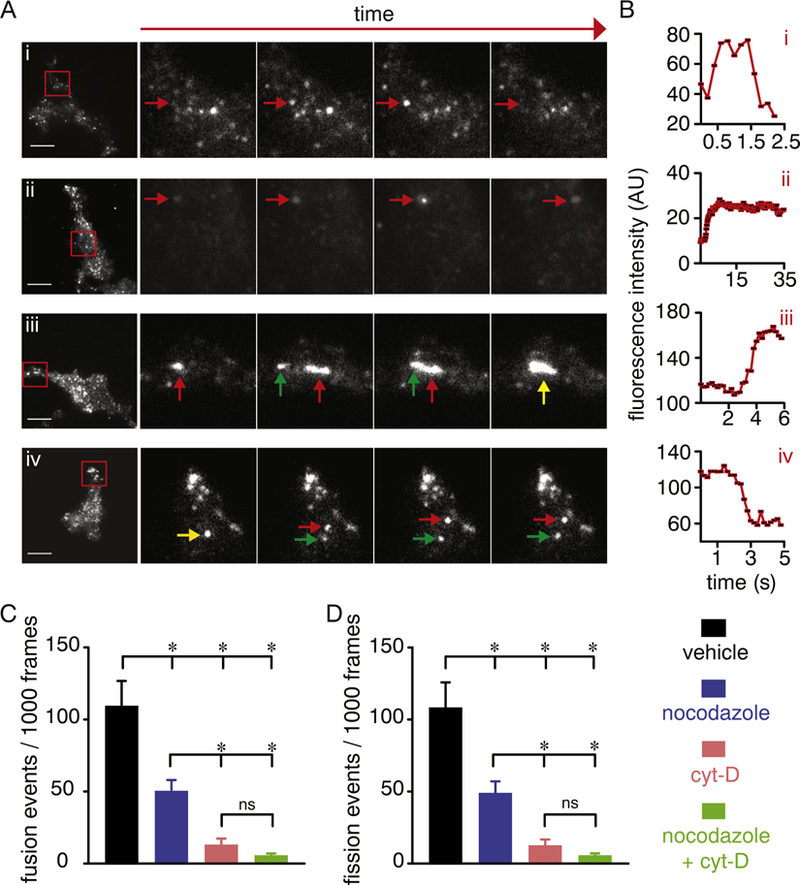
Distinct cytoskeleton-dependent perimembrane behavior of Ca_V_1.2 vesicles. A) Exemplary TIRFM and enlarged time-lapse images of the area highlighted by the red squares in the images on the left side panels showing distinct mobility patterns of Ca_V_1.2-RFP in vehicle-treated tsA-201 cells. Arrows in each image point to the tracking of specific vesicles and their behavior, including “kiss-and-run” (Ai; red arrow), “kiss-and-stay/linger” (Aii; red arrow), “merge-and-linger” (homotypic fusion event; red and green arrows highlight two different vesicles, and the yellow arrow points to fusion of vesicles; Aiii) and “break-and-run” (homotypic fission; yellow arrow highlights a vesicle splitting in two distinct ones highlighted by the red and green arrows; Aiv). Scale bar = 10 μm. B) Time course of mean fluorescence intensity (AU) of Ca_V_1.2 vesicles highlighted by the red arrows in panel A. Bar plots of the frequency of resolvable homotypic fusion (C) and fission (D) events of Ca_V_1.2 vesicles in vehicle-treated (n = 17 cells), 10 μM nocodazole (C; n = 13 cells), 10 μM cytocholasin-D (D; n = 10 cells) or 10 μM nocodazole + cytocholasin-D (E; n = 17 cells) -treated cells. Data are shown as mean ± SEM. *P < 0.05. Kruskal–Wallis test. Significance was compared between data as specified.

Detailed analysis of live-cell TIRFM images of Ca_V_1.2-RFP vesicles revealed distinctive mobility patterns in the perimembrane region in vehicle-treated cells (Fig. 5A and B; Video 12). Many structures exhibited classic “kiss-and-run” behavior, where the Ca_V_1.2 vesicular structure approaches the cell surface and promptly runs away disappearing from the TIRF illumination field within seconds (Fig. 5Ai–Bi; green arrow in Video 12). Other structures displayed “kiss-and-linger” behavior in which the Ca_V_1.2 vesicular structure approached the plasma membrane and stayed there for longer periods of time (Fig. 5Aii–Bii; cyan arrow in Video 12). Intriguingly, Ca_V_1.2 vesicular structures also demonstrated characteristic homotypic fusion and fission events between themselves. Fig. 5Aiii–Biii show an example of two structures fusing with one another (see also Video 13), whereas Fig. 5Aiv and Biv display an example of a large Ca_V_1.2 vesicle splitting in two (see also Video 14). We also noticed examples in which numerous Ca_V_1.2 vesicular structures fused and split from larger ovoid structures (Video 15). Similar results were observed in tsA-201 cells transfected with Ca_V_1.2-GFP (Fig. S6). Depolymerization of microtubules and actin filaments with nocodazole and cyt-D, respectively, significantly reduced the frequency of these fusion and fission events, and simultaneous treatment with both drugs almost completely prevented their occurrence (Fig. 5C and D). These results suggest distinctive mobility patterns for Ca_V_1.2 vesicular structures, which can form unique and dynamic junctions or “hotspots” for Ca_V_1.2 channel interactions with one another via cytoskeleton-dependent fusion and fission events.

### 3.5. Trafficking of Ca_V_1.2 vesicular structure to and from the plasma membrane contributes to Ca_V_1.2 clustering

**Fig. 6.**
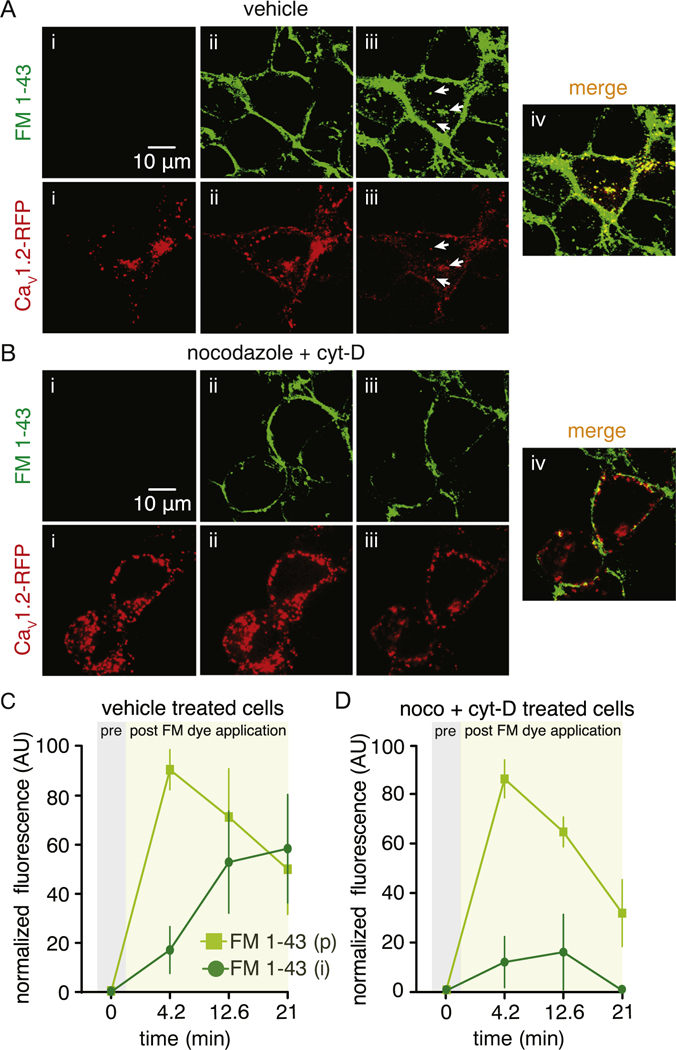
Dynamic interaction of Ca_V_1.2 vesicles with the plasma membrane requires an intact cytoskeleton. Representative dual-color spinning-disk 3D images of Ca_V_1.2-RFP before (i) and after (ii and iii) application of the FM 1–43 dye in vehicle (A) or 10 μM nocodazole + cytocholasin-D (B) -treated cells. White arrows in panel A point to numerous FM 1–43 dye-loaded Ca_V_1.2-RFP-containing vesicles, suggesting active communication between the Ca_V_1.2-RFP structures and the plasma membrane. Plot depicting the time lapse of normalized fluorescence intensity (AU) for the FM 1–43 dye in the perimeter (p) and intracellular (i) side of the cell before and after its application in (C) vehicle or (D) 10 μM nocodazole + cytochalasin-D (n = 6 cells per condition) -treated cells. Data are shown as mean ± SEM.

We examined whether the Ca_V_1.2 vesicular structures undergoing fusion and fission events are physically interacting with the plasma membrane using simultaneous 4D recordings of vehicle-treated Ca_V_1.2-RFP expressing cells with subsequent perfusion of FM 1–43 dye (as above). The large ovoid structures containing Ca_V_1.2 that are characteristic of fusion and fission junctions/hotspots also took up the FM 1–43 dye (Fig. S7) suggesting that these bigger compartments are also in direct communication with the plasma membrane. Interestingly, in the example shown in Fig. S7, the ovoid compartment exhibited complete endocytosis and appeared to divide into smaller distinct endosomes (Video 16). Simultaneous drug-induced depolymerization of microtubules and actin filaments inhibited the dynamic motion of the Ca_V_1.2 vesicular structures and prevented their uptake of FM 1–43 dye (Fig. 6A–D). Quantification of FM 1–43 dye fluorescence at the perimeter (i.e. plasma membrane region) and inside of the cell revealed the efficient uptake of the FM 1–43 dye by the recycling Ca_V_1.2 vesicular structure in vehicle-treated cells (Fig. 6A–D). Conversely, incorporation of FM 1–43 into Ca_V_1.2-containing vesicles was blocked by simultaneous depolymerization of microtubules and actin filaments as evidenced by lack of intracellular Ca_V_1.2 vesicle containing the dye (Fig. 6A–D; Video 17).

**Fig. 7.**
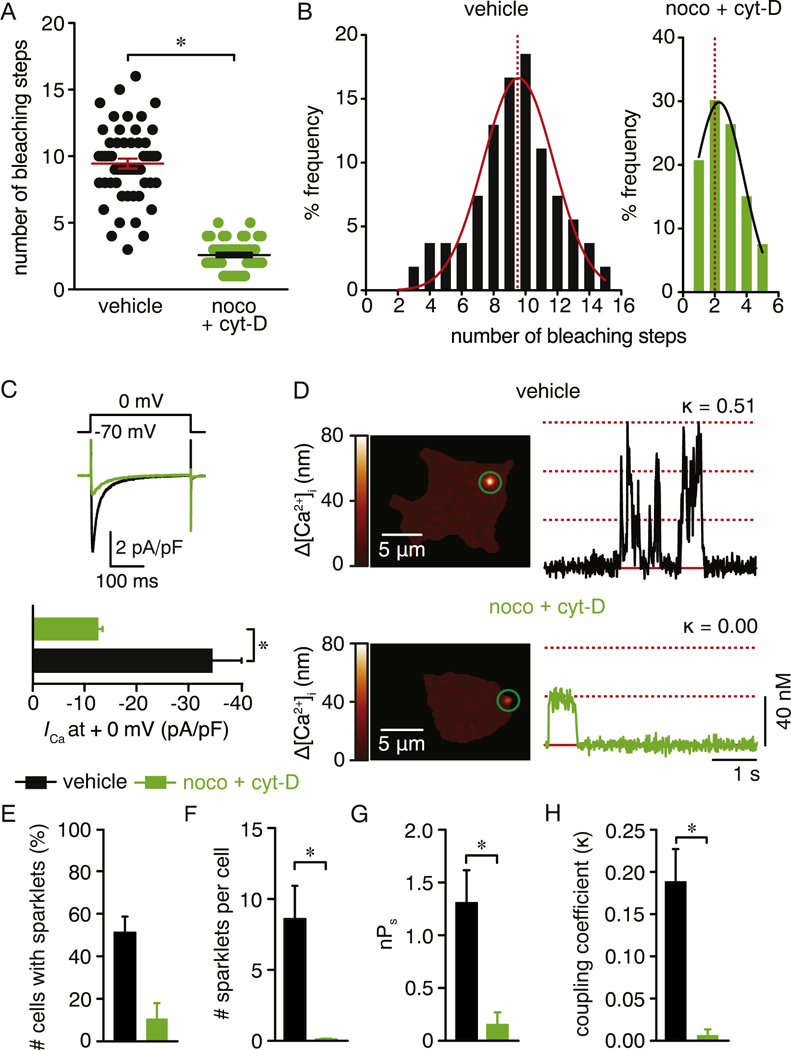
Depolymerization of the cytoskeleton reduces Ca_V_1.2 clustering, channel activity and cooperative gating. A) Scatter plot of the number of bleaching steps obtained from vehicle (n = 54 spots, from 5 cells) and nocodazole + cytocholasin-D (n = 53 spots, from 5 cells) treated cells. Data are shown as mean ± SEM. *P < 0.05. Mann-Whitney test. Significance was compared between data as specified. B) Frequency distribution of bleaching steps between vehicle or nocodazole + cytocholasin-D -treated cells. Histograms were fit using a single Gaussian curve (black lines). The dotted red lines denote the median of the distribution (9 for vehicle-treated cells and 2 for noco + cyt-D-treated cells). C) Representative *I*_Ca_ recording from tsA-201 cells expressing Ca_V_1.2-RFP that were treated with vehicle or 10 μM nocodazole + cytocholasin-D. *I*_Ca_ were evoked by 300 ms depolarization step from a holding potential of −70 mV to 0 mV. Bar plots immediately below summarize the mean ± SEM of the *I*_Ca_ current density at 0 mV. D) Representative TIRFM images showing Ca^2+^ sparklets in cells expressing Ca_V_1.2-RFP that were treated with either vehicle (*upper panel*) or 10 μM nocodazole + cytocholasin-D (*lower panel*). Traces at the right of each image show the time course of [Ca^2+^]_i_ in the respective green circles. k represents the coupling coefficient for each trace. Bar plots showing (E) number of cells with Ca_V_1.2 sparklets, (F) number of Ca_V_1.2 sparklet sites per cell, (G) Ca_V_1.2 sparklet activity and (H) coupling coefficient (k) for Ca_V_1.2 sparklets in vehicle (n = 47 cells) or 10 μM nocodazole + cytocholasin- D (n = 19 cells) -treated cells. Data are shown as mean ± SEM. *P < 0.05. Mann Whitney test. Significance was compared between data as specified.

Trafficking of vesicular Ca_V_1.2 could result in the congregation of Ca_V_1.2 channels in close proximity to one another, ultimately promoting Ca_V_1.2 clustering and the increased probability of their physical interaction; a prerequisite for cooperative gating of Ca_V_1.2 channels [19, 21, 22, 27, 29]. To examine this possibility, we performed TIR-FRAP in vehicle- and nocodazole + cyt-D-treated cells expressing Ca_V_1.2-RFP. Recovery of the Ca_V_1.2-associated fluorescence near the membrane was allowed to proceed for 350 s after which single-particle photobleaching analysis was performed, as before (Fig. S8A). This approach revealed that areas containing Ca_V_1.2 vesicular structures displayed an average of 9 ± 1 discrete photobleaching steps (Fig. 7A–B). Conversely, a significant reduction in Ca_V_1.2-RFP-associated photobleaching steps (2 ± 1) was observed in cells treated with nocodazole + cyt-D (Fig. 7A–B and Fig. S8A). These results suggest that Ca_V_1.2 channels preferentially cluster in structures that are fed by cytoskeleton-dependent trafficking of Ca_V_1.2 vesicles in areas at/near the plasma membrane.

### 3.6. Trafficking of Ca_V_1.2 vesicles contributes to channel activity and cooperative gating of Ca_V_1.2

To test whether dynamic formation and transport of structures containing Ca_V_1.2 contributes to channel activity and cooperative gating, we electrically and optically recorded Ca_V_1.2 activity (e.g. Ca_V_1.2 sparklets) in voltage-clamped tsA-201 cells. In blinded experiments, we found that whole-cell *I*_Ca_ elicited by a depolarizing step from−70 mV to 0 mV, were significantly smaller in cells co-treated with nocodazole + cyt-D compared to vehicle-treated cells (Fig. 7C). Note that cell capacitance and the voltage dependence of activation of Ca_V_1.2 were similar in vehicle-treated cells and cells treated with nocodazole + cyt-D (Fig. S8B–C). The residual current in nocodazole + cyt-D-treated cells is likely the result of Ca_V_1.2 residing already at the plasma membrane at the time of treatment. We also recorded and measured the elementary Ca^2+^ influx events through individual Ca_V_1.2 channels using TIRF microscopy [18, 19, 22]. This is necessary as Ca_V_1.2 activity occurs through distinct foci of heterogeneous activity along the plasma membrane that cannot be easily detected with conventional whole-cell patch-clamp techniques [26, 28]. In blinded experiments, robust Ca_V_1.2 sparklet activity (e.g. nPs; Video 18) was observed in the majority of vehicle-treated cells, which had ~7 Ca_V_1.2 sparklet sites per cell with a quantal unit of Ca^2+^ influx of 38 ± 0.5 nM (Fig. 7D–G and Fig. S8D). Our observations also suggested that a preponderance of the events resulted from cooperative gating of Ca_V_1.2 channels in these cells (Fig. 7H). Quantification of these events with a coupled Markov chain model to assess the strength of cooperative channel gating (coupling coefficient κ; [53]) found that a majority of Ca_V_1.2 sparklets undergo this gating behavior in vehicle-treated cells (Fig. 7H). These observations are in strong agreement with previous results of Ca_V_1.2 sparklets in tsA-201 cells [19, 21, 26, 28, 29, 45]. Co-treatment of Ca_V_1.2-RFP expressing cells with nocodazole and cyt-D significantly reduced Ca_V_1.2 sparklet properties, including cooperative gating behavior and coupling strength (Fig. 7D–H). Together, these results suggest that dynamic trafficking of Ca_V_1.2 vesicles may contribute to Ca_V_1.2 clustering, channel activity and cooperative gating behavior.

## 4. Discussion

In this study, we present evidence that the L-type Ca_V_1.2 channel is trafficked in dynamic vesicles of circular and tubular shape. Notably, these vesicles show distinct patterns of movement and interaction amongst themselves and at the plasma membrane (Fig. S9). These trafficking patterns include homotypic fusion and fission events that are dependent on an intact cytoskeleton. The sites of vesicular homotypic fusion at/near the plasma membrane harbor the highest density of clustered Ca_V_1.2 that appear to sustain L-type calcium channel activity and Ca_V_1.2 sparklet events while facilitating cooperative gating of Ca_V_1.2 channels. Thus, mobile Ca_V_1.2 vesicles undergoing homotypic fusion and fission events may be part of a mechanism that allows tight, dynamic regulation of Ca_V_1.2 function to control Ca^2+^ influx. Such mechanism may have broad implications for modulation of Ca_V_1.2-mediated Ca^2+^ signals in both excitable and non-excitable cells.

Our first major observation is the dynamic movement of Ca_V_1.2 vesicles at/near the membrane. Similar observations were reported in neuronal and pancreatic cell lines expressing Ca_V_1.2 [61, 62], suggesting that this dynamic movement may be a general feature of Ca_V_1.2 channels. Our study revealed that this dynamic movement is mediated via vesicular and tubular compartments. The formation of tubular compartments has been attributed to the function of dynamin GTPase – a universal membrane tubulation protein [63]. These larger tubular compartments could transport more Ca_V_1.2 cargo when compared to vesicular structures [64]. Interestingly, at least four distinctive interaction patterns associated with the dynamic movement of Ca_V_1.2 vesicles were identified. (Fig. S9). These are 1) “kiss-and-run”, in which Ca_V_1.2 vesicles seem to interact transiently with the plasma membrane without fully fusing. 2) Ca_V_1.2 vesicles undergoing “kiss-and-linger/ stay” display transient, yet prolonged dwell-time interactions at/near the plasma membrane. “Kiss-and-run/linger” type of behavior are wellknown alternate modes of communication between protein-containing vesicles, the plasma membrane, and the extracellular space [65, 66]. 3) “Merge-and-linger followed by break-and-run” are mostly observed when rapid homotypic fusion and fission events commence at ovoid compartments at/near the plasma membrane. 4) The ovoid structures may themselves get endocytosed and in the process, may undergo fission into smaller vesicles. A key implication of these observations is that multiple mechanisms may be at work to fine-tune the delivery of Ca_V_1.2 to the plasma membrane.

How do Ca_V_1.2 vesicles interact with the plasma membrane to de-liver functional channels? Classic examples of vesicles fully fusing and collapsing with the plasma membrane show a distinctive and rapid dissipation of the compartment-associated fluorescence [39, 56, 67]. This type of exocytic event, however, was never observed for Ca_V_1.2-containing vesicles, which instead maintained their vesicular nature and never appeared to completely merge with the plasma membrane. Thus, the behavior of Ca_V_1.2-containing vesicles is similar to “kiss-and-run/linger” events previously described [38, 65, 68]. In such a scenario, Ca_V_1.2 could be maintained in a pool of vesicular structures just underneath the plasma membrane for constitutive delivery of channels or alternatively for rapid insertion in response to a stimulus. Indeed, Green et al. showed that in Neuro2a cells expressing Ca_V_1.2 labeled with YFP, Ca_V_1.2-containing vesicles moved rapidly but transiently to the plasma membrane in response to a depolarizing stimulus [61]. Ca_V_1.2 channels may diffuse out of the lingering vesicular structure to the plasma membrane [65, 69] or could remain within the vesicular compartment. Our observation that internalization of Ca_V_1.2 vesicles can still be tracked after their interaction with the plasma membrane suggests that a significant number of Ca_V_1.2-RFP channels are retained within the vesicle. This is supported by the stepwise photobleaching experiments showing a reduced number of bleaching steps in areas of diffused fluorescence compared to areas containing lingering compartments (Fig. 1 and Fig. S1). It is tempting to speculate that the fusion pore formed at the junction of the kissing/lingering compartment and the plasma membrane could provide access of vesicular Ca_V_1.2 to the extracellular space. These channels, which together with plasma membrane-resident Ca_V_1.2, could then respond to different stimuli (e.g. membrane depolarization, G protein coupled receptor signaling, etc.) to promote Ca^2+^ influx [38, 41, 61]. This could represent a mechanism for Ca_V_1.2-mediated diversification of Ca^2+^ signals.

Particularly intriguing was the observation that Ca_V_1.2 vesicles may undergo cytoskeleton-dependent (i.e. microtubules and actin) homotypic fusion and fission events into ovoid compartments. In agreement with previous observations in neurons [24], these types of events may not only dynamically regulate Ca_V_1.2 turnover at/near the plasma membrane, but also promote channel clustering. This possibility is supported by TIR-FRAP - stepwise photobleaching experiments demonstrating that Ca_V_1.2-containing vesicles that linger at/near the plasma membrane have far more Ca_V_1.2 than other areas of the plasma membrane devoid of these lingering ovoid structures (Fig. 7A). From a functional perspective, this clustering may afford the spatial proximity necessary to facilitate the coordinated opening and closing (e.g. co-operative gating) of adjacent Ca_V_1.2 channels, a hypothesis further supported by our findings in recent studies [20, 21, 27, 29]. Indeed, in vehicle-treated cells, a significant number of Ca_V_1.2 sparklet events predominantly exhibited cooperative gating. This was in contrast to observations of events in cells treated with nocodazole and cyt-D where the movement and homotypic fusion of Ca_V_1.2-containing vesicles was halted completely by the drug treatment. Moreover, concomitant depolymerization of microtubules and actin filaments resulted in significantly reduced channel activity and prevention of Ca_V_1.2 co-operative gating (Fig. 7). The dynamic homotypic fusion and fission events of Ca_V_1.2-containing vesicles may also help explain the transient coupling of Ca_V_1.2 channels previously observed in optical and electrical recordings of Ca_V_1.2 expressing cells [20, 21, 27, 29], as opposed to the fully concerted gating observed for other ion channels [70, 71]. Thus, homotypic fusion and fission of lingering Ca_V_1.2 vesicles could be a key mechanism facilitating dynamic Ca_V_1.2 clustering and co-operative gating that could ultimately fine-tune Ca^2+^ signals.

Dynamic Ca_V_1.2 trafficking can be regulated by numerous processes. For example, auxiliary β and α_2_δ subunits are well-known to promote surface expression of Ca_V_1.2 channels in neurons and vascular smooth muscle cells [72–74]. The scaffold protein A-kinase anchoring protein 150 (AKAP150; murine homolog of human AKAP79) has been implicated in Ca_V_1.2 trafficking and regulation, but not in channel clustering in mature neurons [23, 75]. In cardiomyocytes, the adaptor protein bridging integrator 1 (BIN1) seems to play a key role in Ca_V_1.2 trafficking and clustering that is essential for normal cardiac function [76]. In neurons, the actin-binding protein α-actinin [35, 36] and the synaptic protein Densin-180 [77] are associated with Ca_V_1.2 trafficking, Ca_V_1.2 surface retention and signaling. In vascular smooth muscle, the small GTPase protein Rab25 promotes surface expression of Ca_V_1.2 [78]. More recently, the adapter protein Stac was found to regulate trafficking of Ca_V_1.1 channels as well as Ca_V_1.2 activity, which may have important implications for excitation-contraction coupling in skeletal and cardiac muscle, respectively [42, 79]. The phosphorylation state of key residues on the channel also seems to influence Ca_V_1.2 trafficking, dynamics and clustering, at least in neurons [80]. Whether either or all of these proteins and processes are involved in homotypic fusion of Ca_V_1.2 vesicles, channel clustering at specific sites at/near the plasma membrane as well as cooperative gating is currently unknown but are most certainly important questions for future studies. Our findings may shed light on mechanisms of clustering and cooperative gating of other ion channels. For example, the L-type Ca^2+^ channel Ca_V_1.3 [30, 45] as well as the transient receptor potential vanilloid 4 (TRPV4) channel [81, 82] have been shown to cluster at the plasma membrane and undergo cooperative gating events. It is tempting to speculate that homotypic fusion of vesicles containing Ca_V_1.3 or TRPV4 may also contribute to clustering of these channel, which in turn may facilitate their cooperative gating.

Our results also raise a number of intriguing questions. For instance, Does channel activity influence trafficking, clustering and cooperative gating? What are the mechanisms mediating “kiss-and-run” versus “kiss-and-linger” behavior? Are they the result of more stochastic as opposed to highly regulated processes? What are the mechanisms underlying vesicular fusion and fission and how may they contribute to channel clustering and cooperative gating? Are Ca_V_1.2 channels functional while in the vesicular compartment? The use of proteomic approaches applied specifically to Ca_V_1.2-containing vesicles, super-resolution imaging in live cell preparations and genetically-modified mice with fluorescently labeled Ca_V_1.2 will help address these intriguing questions. In conclusion, our work has uncovered an unexpected mechanism for dynamic control of Ca_V_1.2 channels that may be essential for fine-tuning Ca^2+^ influx and cell excitability. Accordingly, homotypic fusion and fission of Ca_V_1.2 vesicles may contribute to functional clustering of Ca_V_1.2 channels to sustain channel activity and transient cooperative gating. Dysregulation of this mechanism may play a role in pathological conditions associated with impaired Ca_V_1.2 function, including neurological disorders [83] and cardiovascular complications such as cardiac arrhythmias and hypertension [19]. This work sets the foundation for future in-depth examinations of the mechanisms underlying dynamic trafficking of Ca_V_1.2 during physiological and pathological conditions.

## Supplementary Material

Supplement

Video 17

Video 18

Video 2

Video 3

Video 4

Video 5

Video 6

Video 7

Video 8

Video 9

Video 1

Video 10

Video 11

Video 12

Video 13

Video 14

Video 15

Video 16

## Abbreviations:

TIRFM: Total Internal Reflection Fluorescence Microscopy
tRFP: tag Red Fluorescence Protein
FBS: fetal bovine serum
Noco: nocodazole
Cyt-D: cytochalasin D
TIR-FRAP: Total Internal Reflection – Fluorescence Recovery after Photobleaching
MSD: mean square displacement
FWHM: full-width at half maximum
ROI: region of interest
Ca^2+^: calcium
Ba^2+^: barium
ANOVA: One-way analysis of variance
PKCα: protein kinase C α
