# Supplementary material for: Dynamic L-type CaV1.2 channel trafficking facilitates CaV1.2 clustering and cooperative gating
Source: Biochim Biophys Acta Mol Cell Res. Author manuscript; Available in PMC 2019 Mar 8. (PMC6407617; doi:10.1016/j.bbamcr.2018.06.013)
Supplement: Supplement [file NIHMS993014-supplement-Supplement.docx]

**Supplementary Information**

**Dynamic L-type Ca_V_1.2 channel trafficking facilitates Ca_V_1.2 clustering and cooperative gating**

Debapriya Ghosh^a^, Madeline Nieves-Cintrón^a^, Sendoa Tajada^b^, Ingrid Brust-Mascher^c^, Mary C. Horne^a^, Johannes W. Hell^a^, Rose E. Dixon^b^, Luis F. Santana^b^ and Manuel F. Navedo^a*^

^a^Department of Pharmacology and ^b^Department of Physiology & Membrane Biology, School of Medicine, and ^c^Advanced Imaging Facility, School of Veterinary Medicine, One Shields Avenue, University of California, Davis, CA 95616, USA

*Corresponding and Lead Contact Author:

Manuel F. Navedo, PhD

Department of Pharmacology

University of California, Davis

One Shields Avenue, Davis, CA 95616

email: [mfnavedo@ucdavis.edu](mailto:mfnavedo@ucdavis.edu)

Tel. 530-752-6880

Fax. 530-752-7710

**
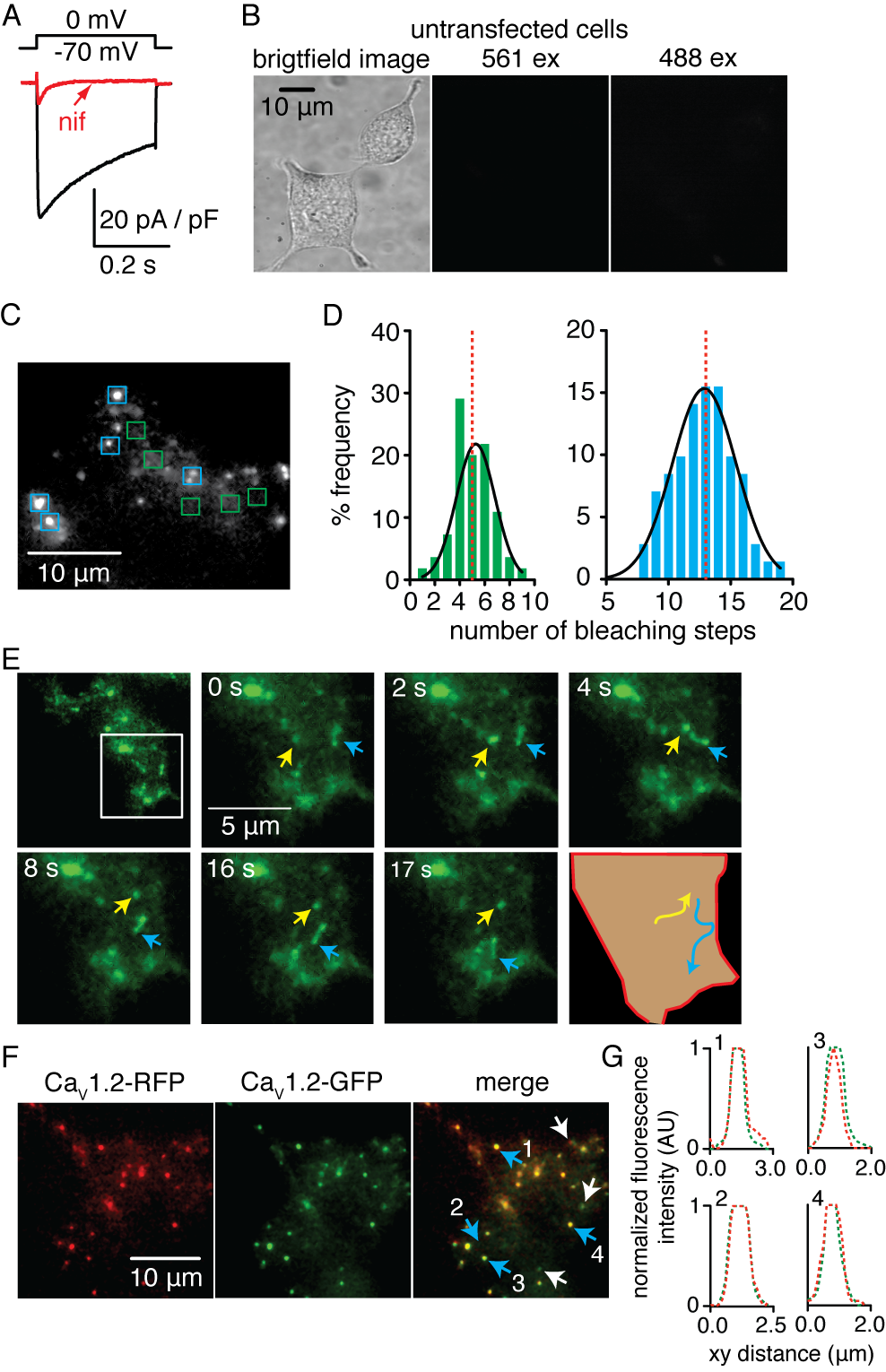
Supplemental Figure Legends**

**Figure S1 related to Figure 1: Robust nifedipine-sensitive *I*_Ba_ in cells transfected with Ca_V_1.2, and heterogeneous distribution and dynamic perimembrane movement of Ca_V_1.2-GFP as well as of cells co-transfected with Ca_V_1.2-RFP and Ca_V_1.2-GFP. A)** Representative *I*_Ba_ recording from tsA-201 cells expressing Ca_V_1.2 under control condition and after application of the L-type Ca^2+^ channel blocker nifedipine (200 nM). *I*_Ba_ were evoked by 300 ms depolarization step from a holding potential of -70 mV to 0 mV (n = 6 cells). **B)** Representative TIRFM images of untransfected cells acquired in brightfield, and with 561 and 488 laser excitation (n = 5 cells). **C)** Representative TIRFM image of a fixed cell expressing Ca_V_1.2-GFP. The blue and green squares highlight areas with clearly identifiable Ca_V_1.2 structures and with diffuse fluorescence (DF), respectively, that were used for analysis. **D)** Frequency distribution of bleaching steps between DF (green histogram) and structure (blue histogram) regions. Histograms were fit using a single Gaussian curve (black lines). The dotted red lines denote the median of the distribution (DF = 5; n = 55 regions from 4 cells. Structures = 13; n = 71 regions from 4 cells). **E)** TIRFM images at consecutive intervals depicting the perimembrane movement of Ca_V_1.2 structures in vehicle-treated cells transfected with Ca_V_1.2-GFP. Arrowheads point at the track of different Ca_V_1.2 structures. The *lower right corner* image illustrates the movement tracks of the respective structures highlighted by the arrowheads. **F)** TIRFM images of cells co-transfected with Ca_V_1.2-RFP and Ca_V_1.2-GFP (n = 5 cells). Blue arrows in the merged image illustrate Ca_V_1.2 structures in which both Ca_V_1.2-RFP and Ca_V_1.2-GFP seem to co-exist, whereas white arrows highlight other areas that seem to contain only Ca_V_1.2-RFP or Ca_V_1.2-GFP. These data suggest that dynamic movement of Ca_V_1.2 structures is an intrinsic property of Ca_V_1.2 channels, and not the result of artifacts due to over-expression or detachment of the tagged fluorescence protein. **G)** Line profile plots of the RFP- and GFP-associated fluorescence for the Ca_V_1.2 structures numbered in the merged image in panel F.

**
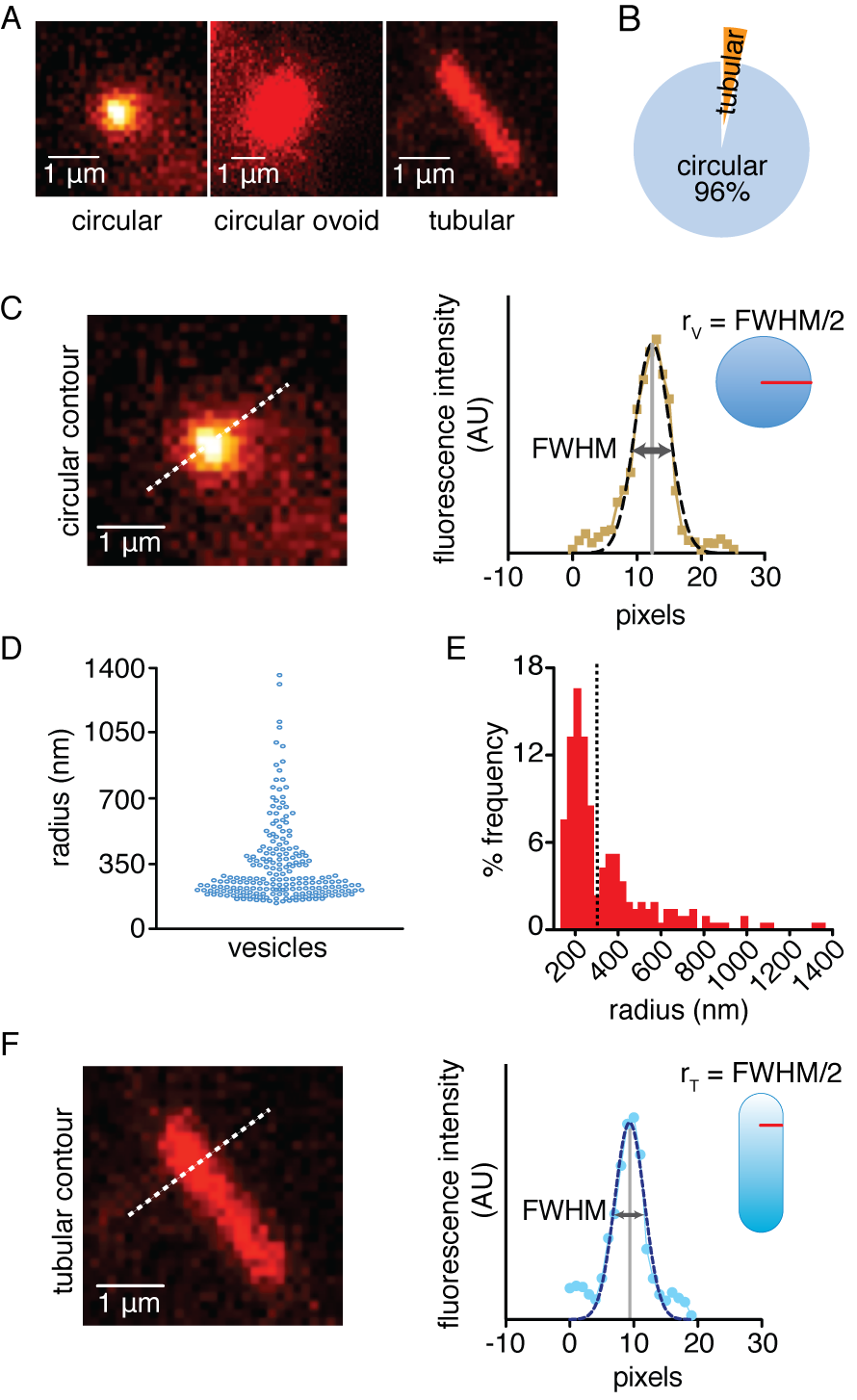
**

**Figure S2 related to Figure 1: Morphological analysis of Ca_V_1.2 vesicular structures. A)** Representative TIRFM images of circular and tubular structures containing Ca_V_1.2-RFP in vehicle-treated cells. **B)** Pie chart quantifying the proportion of circular and tubular structures containing Ca_V_1.2 (n = 12 cells, 962 structures). **C)** Representative TIRFM image of a circular structure containing Ca_V_1.2-RFP and corresponding fluorescent profile (light brown squares) of the white dotted line. The fluorescent profile of this particular structure was fitted with a Gaussian curve (black dotted line) to obtain the expected width for each structure measured as the full-width-at-half-maximum (FWHM). The radius ‘r_V_’ was obtained by dividing FWHM by 2. **D)** Scatter plot and **E)** frequency histogram illustrating the spread and frequency distribution, respectively, of the radius of circular structure (n = 11 cells, 211 vesicles). The dotted line at ~300 nm in the frequency distribution histogram reflects the boundary between the populations of structures with clearly distinct radius sizes. **F)** Exemplary TIRFM image of a tubular structure and corresponding fluorescent profile (light blue circles) of the white dotted line. The fluorescent profile of this particular tubule was fitted with a Gaussian curve (dark blue dotted line) to obtain the expected width for each tubule measured as the full-width-at-half-maximum (FWHM). The radius ‘r_T_’ was obtained by dividing FWHM by 2 (n = 9 cells, 16 tubules).

**
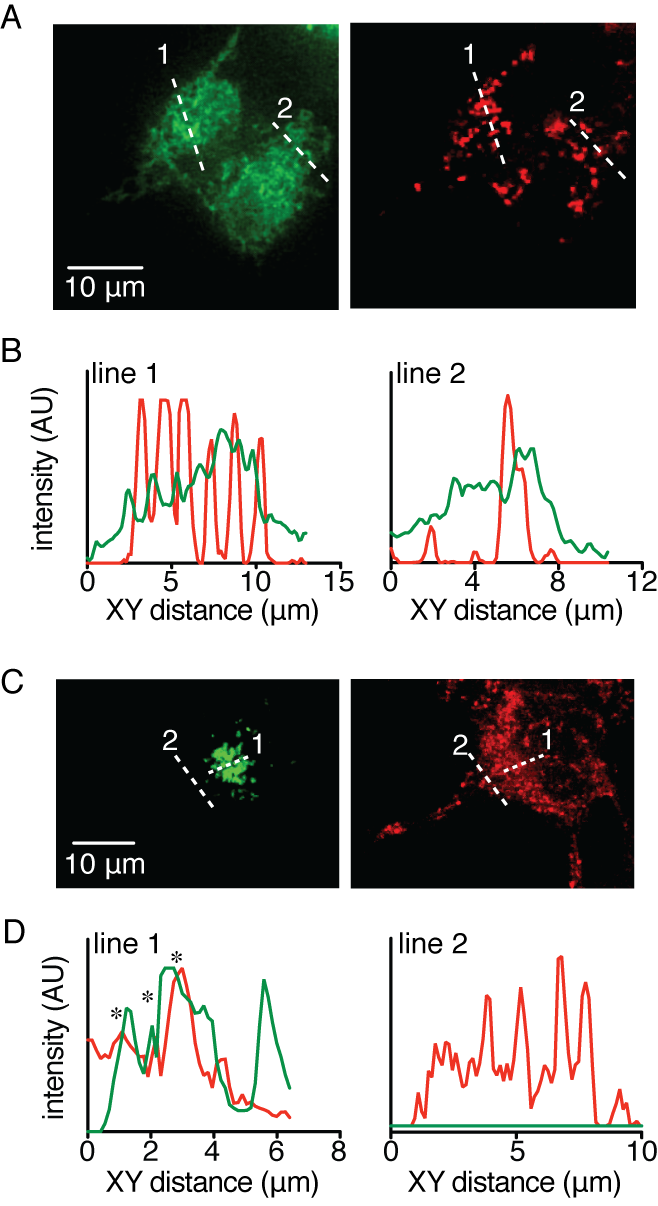
**

**Figure S3 related to Figure 2: Ca_V_1.2 vesicles are Golgi-derived, but independent from both Golgi and endoplasmic reticulum compartments. A)** Dual-color 4D recording of a cell expressing Ca_V_1.2-RFP and the general ER marker Sec61β-GFP. **B)** The fluorescent intensity profile of Ca_V_1.2-RFP- and Sec61β-GFP was measured along the dotted lines in region 1 and 2 in A. **C)** Dual color 4D recording of a cell expressing Ca_V_1.2-RFP and the Golgi resident protein glycosyltransferases-GFP. The location of the Golgi body near the central part of the cell is apparent. **D)** The fluorescent intensity profile of Ca_V_1.2-RFP- and glycosyltransferases-GFP was measured along the dotted lines in region 1 and 2 in C. The asterisks over the profile of line 1 indicate areas of apparent colocalization of GFP and RFP signals. Note that in region 2, only the Ca_V_1.2-RFP-associated fluorescence was observed. Experiments were repeated in 5 independent cells per condition.

**
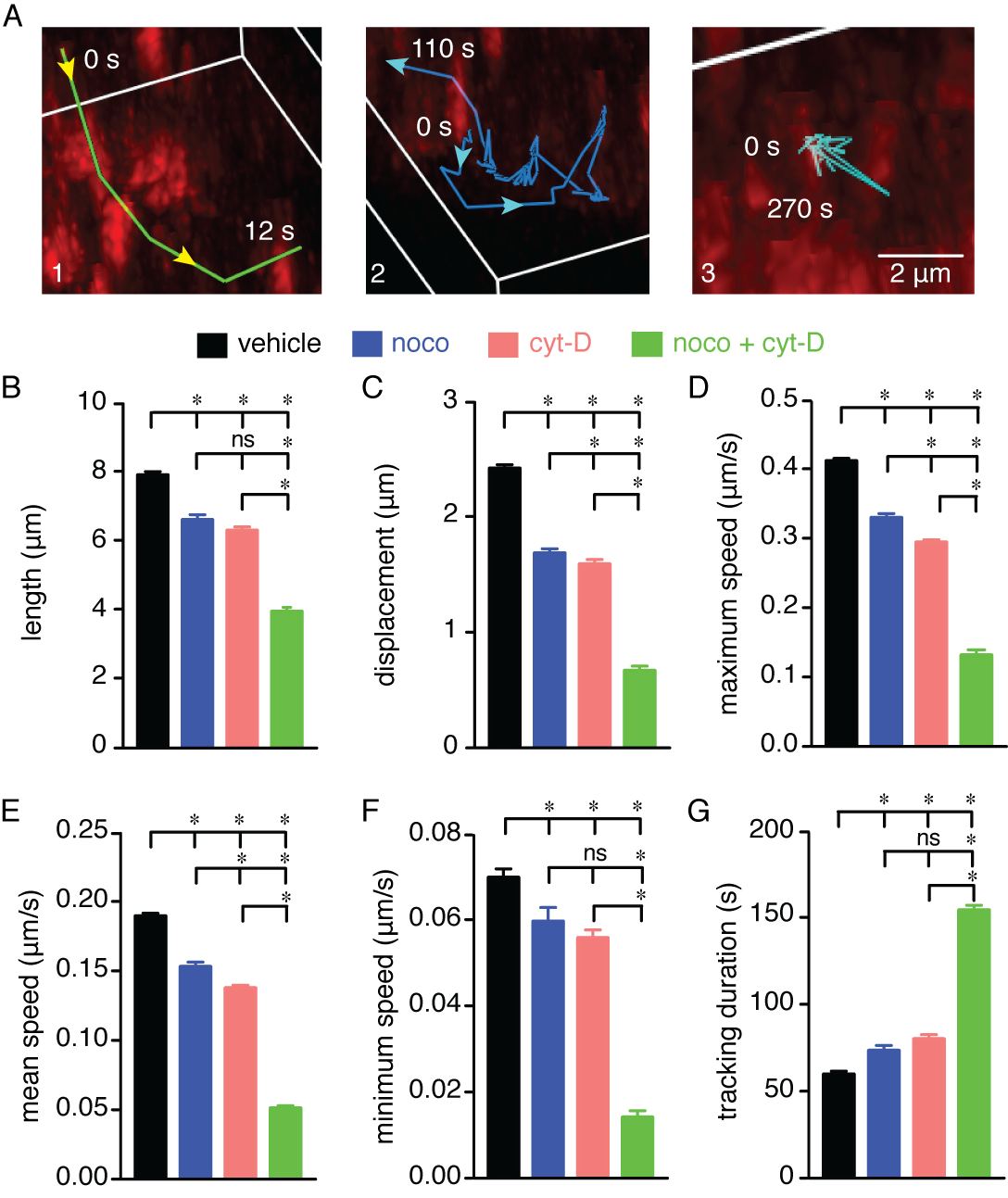
**

**Figure S4 related to Figure 3: Properties of mobile Ca_V_1.2 vesicles. A)** Enlarged 3D images of sections depicting trajectories 1, 2 and 3 in Figure 2B. Amalgamated data of the (**B**) length, (**C**) displacement, (**D**) maximum speed, (**E**) mean speed, (**F**) minimum speed and (**G**) duration of Ca_V_1.2 vesicles in vehicle (n = 19 cells, 5480 vesicles), nocodazole (10 μM; n = 12 cells, 1891 vesicles), cytochalasin-D (10 μM; n = 14 cells, 4227 vesicles) or nocodazole and cytochalasin-D (10 μM each; n = 7 cells, 1405 vesicles) -treated cells. Data are shown as mean ± SEM. **P* < 0.05. Kruskal–Wallis test. Significance was compared between data as specified.

**
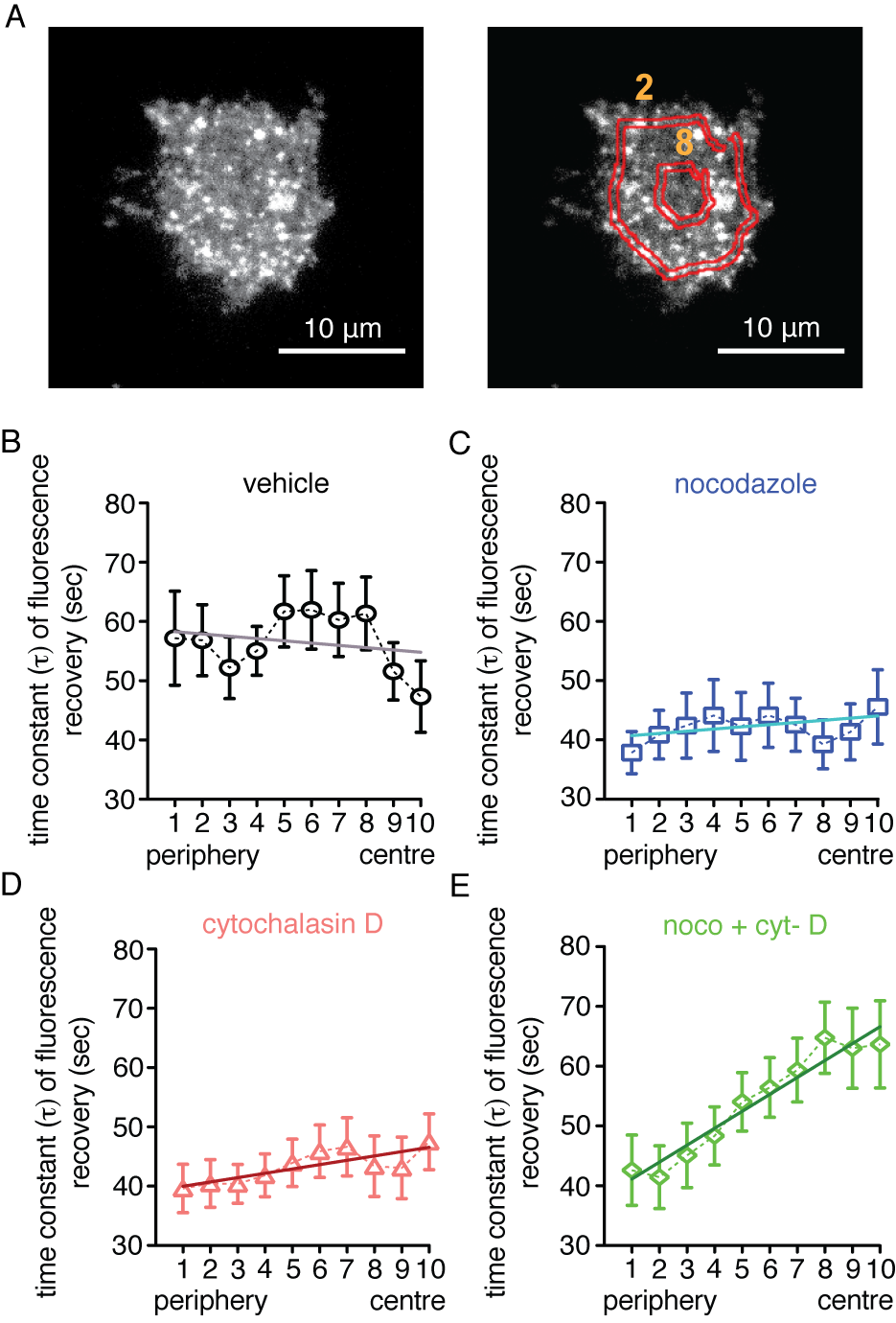
**

**Figure S5 related to Figure 4: Analysis of Ca_V_1.2-RFP mobility post photobleaching. A)** Representative TIRFM images depicting the footprint of a vehicle-treated tsA-201 cell expressing Ca_V_1.2-RFP. The *right-side panel* displays two superimposed concentric bands that were used for analysis at the outer (2) and inner (8) zone. Exponential time constants for the fluorescent recovery in the 10 concentric bands analyzed in vehicle (**B**; n = 15 cells), 10 μM nocodazole (**C**; n = 10 cells), 10 μM cytocholasin-D (**D**; n = 10 cells) or 10 μM nocodazole + cytocholasin-D (**E**; n = 17 cells) -treated cells. Data are shown as mean ± SEM.

**
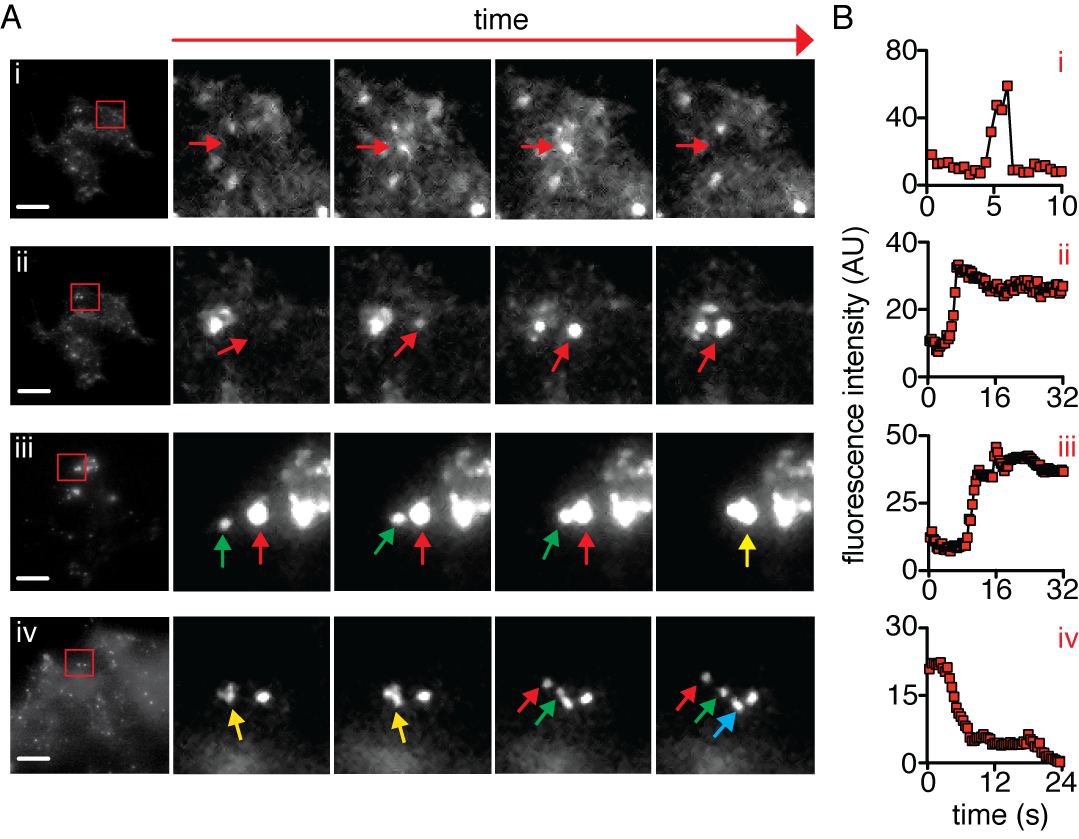
**

**Figure S6 related to Figure 5: Ca_V_1.2-GFP vesicles exhibit similar cytoskeleton-dependent perimembrane behavior as Ca_V_1.2-RFP vesicles. A)** Exemplary TIRFM and enlarged time-lapse images of the area highlighted by the red square in the left side panels showing distinct mobility patterns of Ca_V_1.2-GFP in vehicle-treated tsA-201 cells. Arrows in the images point to the tracking of specific vesicles and their behavior, including “kiss-and-run” (**Ai**; red arrow), “kiss-and-stay/linger” (**Aii**; red arrow), “merge-and-linger” (homotypic fusion event; red and green arrows highlight two different vesicles, and the yellow arrow points to fusion of vesicles; **Aiii**) and “break-and-run” (homotypic fission; the yellow arrow highlight a vesicle that is splitting in three as highlighted by the red, green and blue arrows; **Aiv**). Scale bar = 10 µm. **B)** Mean fluorescence time course of Ca_V_1.2 vesicles highlighted by the red arrows in panel A for each behavior.

**
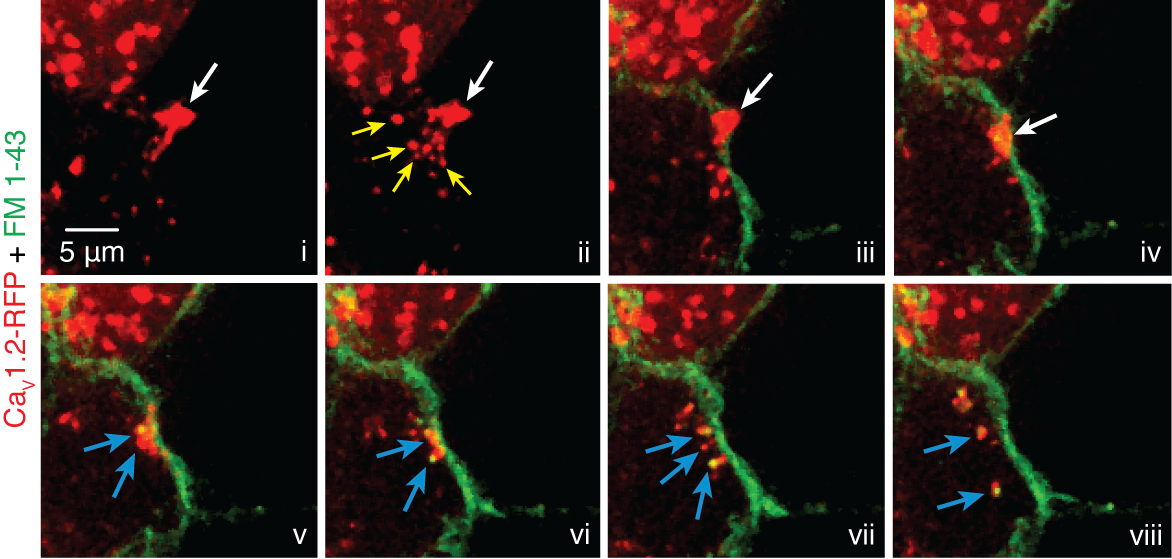
**

**Figure S7 related to Figure 6: Dynamic homotypic fusion and fission events of Ca_V_1.2 vesicles at /near the plasma membrane.** Representative dual-color spinning-disk 3D images of a vehicle-treated cell expressing Ca_V_1.2-RFP depicting the position of an ovoid homotypic fusion site (white arrow) before and after application of the FM 1-43 dye. Images i-iv highlight areas of active fusion events with the ovoid junction at/near the membrane, as illustrated by the loading of the Ca_V_1.2 vesicles with the FM 1-43 dye (yellow structures). Subsequently, images in v-viii show that this large vesicular structure gets internalized and breaks down into smaller units (blue arrows) illustrating “break and run” behavior.

**
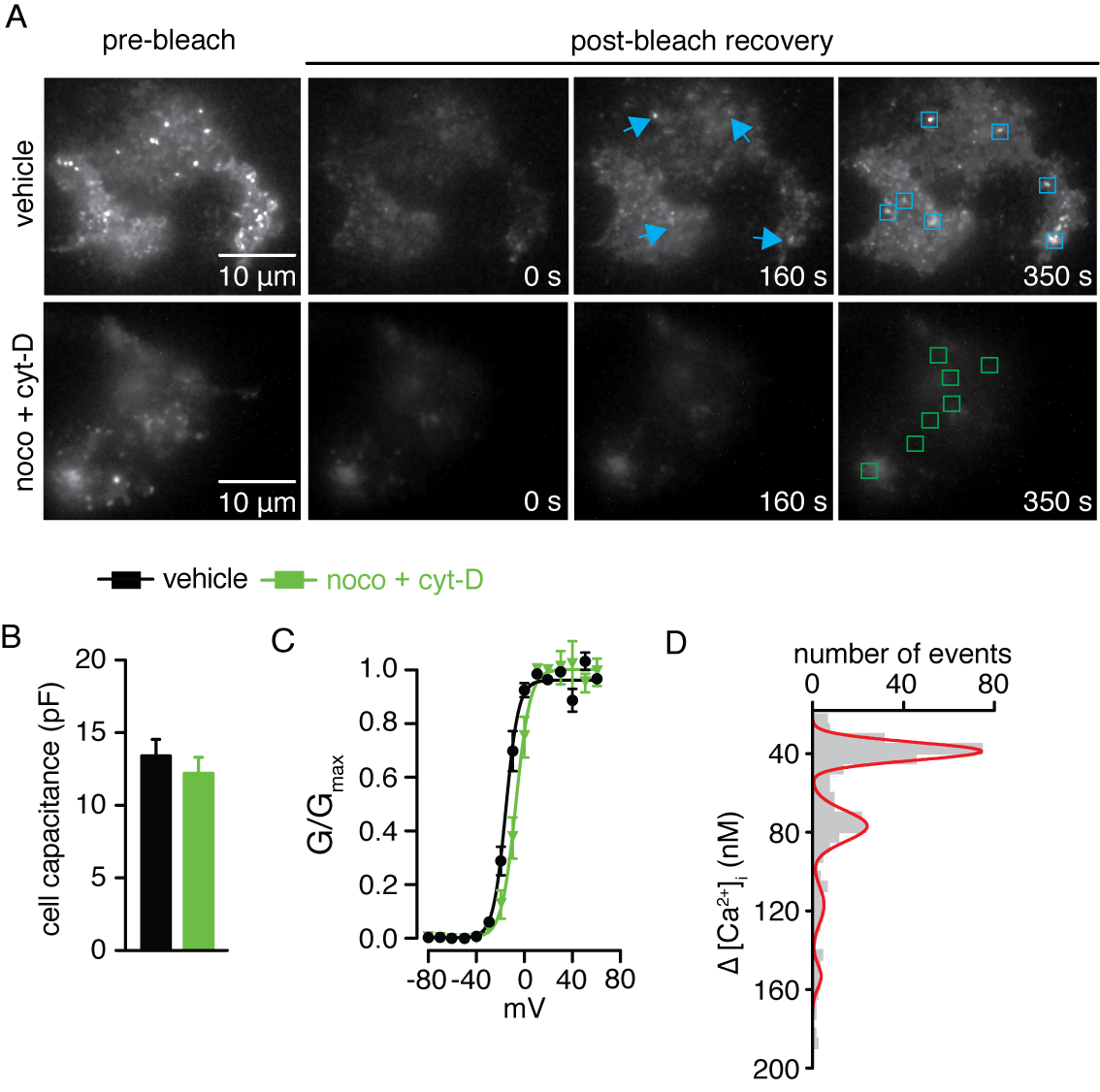
**

**Figure S8 related to Figure 7: Stepwise bleaching experiments and biophysical properties of Ca_V_1.2. A)** Representative TIRFM image of cells expressing Ca_V_1.2-RFP treated either with vehicle or 10 μM nocodazole + cytocholasin-D. Cells were fixed after TIR-FRAP was completed. The blue arrows highlight areas of robust recovery of Ca_V_1.2 in vehicle treated cell. The blue and green squares highlight areas that were used for analysis. **B)** Bar plots summarizing the mean ± SEM of the cell capacitance in vehicle (n = 12 cells) or 10 μM nocodazole + cytocholasin-D (n = 9 cells) -treated cells. **C)** Voltage-dependence of activation of *I*_Ca_ is shown as a plot of normalized G/G_max_ in cells treated with vehicle (n = 12 cells) or 10 μM nocodazole + cytocholasin-D (n = 9 cells). Smooth lines represent best-fit curves to the data with a least-squared method using a Boltzmann equation. Mann-Whitney test with *P* < 0.05 for significance. **D)** Amplitude histogram of Ca_V_1.2 sparklets with 20 mM external Ca^2+^ in vehicle-treated cells expressing Ca_V_1.2-RFP (n = 47 cells). The solid red line is the best fit to the data following a multi-component Gaussian function.

**
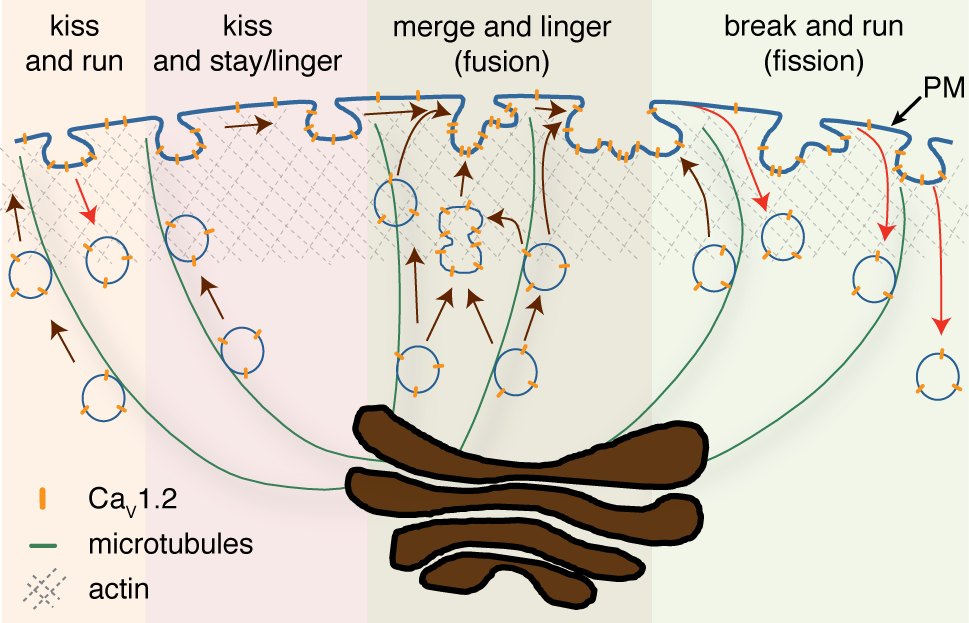
**

**Figure S9: Model for dynamic trafficking of Ca_V_1.2 regulating channel clustering, activity, and cooperative gating.** Cartoon depicting different types of trafficking behavior of Ca_V_1.2 compartments at/near the plasma membrane. During “kiss and run”, Ca_V_1.2-containing vesicles come at/near the membrane and after a brief lingering period are endocytosed. In “kiss and stay/lingering”, compartments come at/near the membrane and remain there for a prolonged period of time before being endocytosed or merged into bigger vesicular compartments, as in the case of homotypic fusion events. These fusion events are characterized by fusion of two or more distinct Ca_V_1.2-containing vesicles at/near the plasma membrane. These areas of homotypic fusion harbor a larger number of Ca_V_1.2 channels than other areas of the plasma membrane but can also undergo fission events in which the larger structure breaks down into smaller structures that seem to be endocytosed. These types of Ca_V_1.2 trafficking patterns are very dynamic and transient processes that sustain Ca_V_1.2 clustering, channel activity and cooperative gating, which may have major implications in cell function.

**Supplemental Video Legends**

**Video 1:** TIRFM movie showing constitutive near membrane (~100 nm) movement of Ca_V_1.2-containing structures in a vehicle-treated tsA-201 cell. Video was generated at 20 frames per second.

**Video 2:** 4D confocal movie showing movements of Ca_V_1.2-containing structures (Ca_V_1.2-RFP – red) before and after application of the plasma membrane dye FM 1-43 (green). Scale bar = 10 μm. Video was generated at 20 frames per second.

**Video 3:** 4D confocal movie showing the constitutive intracellular movement of Ca_V_1.2 structures in a vehicle-treated tsA-201 cell. Video was generated at 20 frames per second.

**Video 4**: 4D confocal movies showing the 561 channel (*left panel*), the 488 channel (*center panel*) and merge images (*right panel*) of two vehicle-treated, untransfected tsA-201 cells (green and red arrows) next to a cell that was transfected with Ca_V_1.2-RFP (red) before and after application of the plasma membrane dye FM 1-43 (green). Note that untransfected cells do not have apparent Ca_V_1.2-RFP structures. Video was generated at 20 frames per second.

**Video 5:** 4D confocal movie showing the movement of Ca_V_1.2-RFP and the ER marker sec61β-GFP in a tsA-201 cell. Scale bar = 10 μm. Video was generated at 20 frames per second.

**Video 6:** 4D confocal movie showing the movement of Ca_V_1.2-RFP and the Golgi marker glycosyltransferases-GFP in a tsA-201 cell. Scale bar = 10 μm. Video was generated at 20 frames per second.

**Video 7:** 4D confocal movie showing movements of Ca_V_1.2 vesicular structures (Ca_V_1.2-RFP – red) to and from the plasma membrane (FM 1-43 dye – green) where the recycling Ca_V_1.2 vesicles rapidly take up the FM 1-43 dye. Scale bar = 10 μm. Video was generated at 20 frames per second.

**Video 8:** 4D confocal movie showing the intracellular movement of Ca_V_1.2 vesicular structures in a tsA-201 cell treated with 10 μM nocodazole. Video was generated at 20 frames per second.

**Video 9:** 4D confocal movie showing the intracellular movement of Ca_V_1.2 vesicular structures in a tsA-201 cell treated with 10 μM cytochalasin-D. Video was generated at 20 frames per second.

**Video 10:** 4D confocal movie showing the intracellular movement of Ca_V_1.2 vesicles in a tsA-201 cell treated with 10 μM nocodazole + 10 μM cytochalasin-D. Video was generated at 20 frames per second.

**Video 11**: TIRFM movie showing constitutive near membrane (~100 nm) movement of Ca_V_1.2 vsicles in a vehicle-treated tsA-201 cell following Ca_V_1.2-RFP-associated fluorescence recovery after photobleaching of the evanescent field. Video was generated at 20 frames per second.

**Video 12:** TIRFM movie showing an example of “kiss and run” (green arrow) and “kiss and stay/linger” (blue arrow) behavior of Ca_V_1.2 vesicles at/near the plasma membrane. Scale bar = 2 μm. Video was generated at 20 frames per second.

**Video 13:** TIRFM movie showing an example of fusion of individual Ca_V_1.2 vesicular structures at/near the plasma membrane. Scale bar = 2 μm. Video was generated at 20 frames per second.

**Video 14:** TIRFM movie showing an example of fission of a larger Ca_V_1.2 vesicular structure into separate individual Ca_V_1.2-containing structures at/near the plasma membrane. Scale bar = 2 μm. Video was generated at 20 frames per second.

**Video 15:** TIRFM movie showing an example of fusion and fission of multiple Ca_V_1.2 vesicles from a central bigger ovoid junctional structure (blue arrowhead) at/near the plasma membrane. Scale bar = 2 μm. Video was generated at 20 frames per second.

**Video 16:** 4D confocal movie showing movements of a Ca_V_1.2 vesicular fusion/fission junction (blue arrow) residing at the plasma membrane (FM 1-43 dye – green) as evident by rapid uptake of the FM 1-43 dye by the structure, which eventually gets endocytosed and breaks down into several smaller Ca_V_1.2-containing structures. Scale bar = 5 μm. Video was generated at 20 frames per second.

**Video 17:** 4D confocal movie showing the effects of cytoskeleton depolymerization on Ca_V_1.2 vesicle dynamics in the presence of the plasma membrane dye FM 1-43. Scale bar = 10 μm. Video was generated at 20 frames per second.

**Video 18:** Ca_V_1.2 sparklet in tsA-201 cells treated with vehicle and after treatment with nocodazole and cytochalasin-D.
